# Molecular Response of *Ulva prolifera* to Short-Term High Light Stress Revealed by a Multi-Omics Approach

**DOI:** 10.3390/biology11111563

**Published:** 2022-10-25

**Authors:** Kai Gu, Yuling Liu, Ting Jiang, Chuner Cai, Hui Zhao, Xuanhong Liu, Peimin He

**Affiliations:** 1 College of Marine Ecology and Environment, Shanghai Ocean University, Shanghai 201306, China; 2 National Demonstration Center for Experimental Fisheries Science Education, Shanghai Ocean University, Shanghai 201306, China; 3 Co-Innovation Center of Jiangsu Marine Bio-industry Technology, Lianyungang 222005, China

**Keywords:** *Ulva prolifera*, light stress, transcriptomics, proteomics, metabolomics, lipidomics

## Abstract

**Simple Summary:**

High light stress is one of the main factors affecting the normal growth of *Ulva prolifera*. The response mechanism of *U. prolifera* to 12 h of high light stress was explored by the multi-omics method. We found that short-term high light could inhibit the assimilation process of *U. prolifera*, destroy the cellular structure, and inhibit respiration. Moreover, it was raised by the genes associated with photosynthetic pigment synthesis, optical system I, and electronic transport, and may be able to make up the ATP defects by circulating electronic transport. At the same time, it reduced NADPH production by attenuating photosystem II synthesis. The carbon fixed approach was also transformed from the C3 pathway to the C4 pathway. Revealing the response mechanism of *U. prolifera* to high light can provide a more theoretical basis for studying the outbreak of green tide of *U. prolifera* in the Yellow Sea.

**Abstract:**

The main algal species of *Ulva prolifera* green tide in the coastal areas of China are four species, but after reaching the coast of Qingdao, *U. prolifera* becomes the dominant species, where the light intensity is one of the most important influencing factors. In order to explore the effects of short-term high light stress on the internal molecular level of cells and its coping mechanism, the transcriptome, proteome, metabolome, and lipid data of *U. prolifera* were collected. The algae were cultivated in high light environment conditions (400 μmol·m^−2^·s^−1^) for 12 h and measured, and the data with greater relative difference (*p* < 0.05) were selected, then analyzed with the KEGG pathway. The results showed that the high light stress inhibited the assimilation of *U. prolifera*, destroyed the cell structure, and arrested its growth and development. Cells entered the emergency defense state, the TCA cycle was weakened, and the energy consumption processes such as DNA activation, RNA transcription, protein synthesis and degradation, and lipid alienation were inhibited. A gradual increase in the proportion of the C4 pathway was recorded. This study showed that *U. prolifera* can reduce the reactive oxygen species produced by high light stress, inhibit respiration, and reduce the generation of NADPH. At the same time, the C3 pathway began to change to the C4 pathway which consumed more energy. Moreover, this research provides the basis for the study of algae coping with high light stress.

## 1. Introduction

From 2007 to 2020, large-scale green tide disasters occurred in the Yellow Sea in China [1], which severely affected the ecosystem and the lives of coastal residents in the sea area. Green tides are ecological anomaly caused by a sharp increase in green algae’s biomass, and *U. prolifera* has always been a species in the outbreak of green tide algae in the Yellow Sea [2]. *U. prolifera* (*Ulvales*, *Chlorophyta* [1,3]) has strong resistance to the complex environmental conditions in the sea. It can also resist and adapt to the increasing light intensity in the Yellow Sea from spring to summer during floating and can reproduce rapidly [4]. *U. prolifera* reaches the Shandong Peninsula and Qingdao coastal waters in July and becomes the only surviving species.

The required light intensity for algae growth is generally between 33 and 400 μmol·m^−2^·s^−1^ [5], and the change of light intensity has different effects on its growth, photosynthesis, and respiration [6]. For *U. prolifera*, 40 μmol·m^−2^·s^−1^ is the lowest required light intensity for algae growth, and 60–140 μmol·m^−2^·s^−1^ are the optimal values (the light intensity range in the Yellow Sea area in May and June), with the algae reaching the highest daily growth rate [5]. With the increased light intensity after July, the growth of other early component species of green tide, including *Ulva linza*, *Ulva compressa*, and *Ulva flexuosa*, is inhibited at 160 μmol·m^−2^·s^−1^. However, the instantaneous net photosynthetic performance of *U. prolifera* increases significantly at 160 μmol·m^−2^·s^−1^, and the relative growth rate at 280 μmol·m^−2^·s^−1^ is even higher than that under low light conditions [7,8]. *U. prolifera* can even survive under 200–600 μmol·m^−2^·s^−1^ [9], showing that it has strong tolerance to high light intensity, and the mechanism is worth studying.

With the rapid development of high-throughput sequencing techniques, omics technology has become the mainstream for studying organisms’ responses to the environment. Jia et al. obtained many expressed sequence tags of *U. prolifera*, marking the parts that may contribute to the rapid growth of algae [10]. The comparative transcriptome of *U. prolifera*, *U. linza*, *U. flexuosa*, and *U. compressa* showed the difference in the construction of transcription factors and metabolic pathways of *U. prolifera*, as well as the enrichment of pyruvate kinase and nitrate transporters in these growth-related genes [11]. However, the current research on the effects of environmental factors on *U. prolifera* has focused on the temperature stress. For example, the transcriptome has been used to identify the relevant genes of *U. prolifera* involved in the carotenoid biosynthesis pathway at different temperatures [12] and proteomics has been used to study the changes in the protein expression of *U. prolifera* at high temperatures [13]. The research on the response to light intensity has focused on physiological ecology or individual genes, e.g., the ELIP-like genes in *U. linza* may be involved in photoprotection under high light, and low temperature and low osmotic stress. Therefore, there is no comprehensive and systematic understanding of the molecular mechanism of *U. prolifera* responding to light intensity.

The previous transcriptomics studies have shown that the above four green tide algae have significant differences in response to different light intensities and provide a reference for establishing the light intensity models. On this basis, the work combined the transcriptome, proteome, metabolome, and lipidome to study the environmental response of *U. prolifera* under high light intensity, thus revealing the biological mechanism of *U. prolifera*.

## 2. Materials and Methods

### 2.1. Materials

*U. prolifera* samples were collected from Qingdao waters (120°19′ E, 36°04′ N) in July 2008, and gametophytes’ pure-line progeny were obtained through sterile subculturing in the laboratory. In this experiment, samples of *U. prolifera* gametophytes were subcultured in VSE medium at 20 °C, a light intensity of 120 μmol·m^−2^·s^−1^, and light period/dark period = 12:12 h. After 15 days of cultivation, the algae with healthy growth and similar morphologies were taken. The experiment was divided into the high light intensity treatment (400 μmol·m^−2^·s^−1^) and the control group (120 μmol·m^−2^·s^−1^), with other conditions unchanged. After the two groups were cultured for 12 h, the algae were taken out immediately. After liquid nitrogen treatment and ultra-low-temperature freeze-drying, omics tests were performed separately. The experiment set up biological replicates, where the transcriptome and proteome had three replicates per group, and the metabolome and lipidome had six per group. Each of the above replicates contained ten fronds.

### 2.2. Transcriptomics Procedure

In the transcriptome experiment, the total RNA from *U. prolifera* samples was accurately quantified after extracting. mRNA capture and fragmentation were performed. After the first strand was synthesized, double-strand cDNA synthesis was performed. Subsequently, the library was amplified with quality testing, and the obtained cDNA library was subjected to high-throughput sequencing on Illumina Hiseq TM (Illumina Inc., San Diego, CA, USA). Then, fast quality control quality evaluation was performed on the original sequencing data, and the quality was cut by Trimmomatic to obtain relatively accurate and valid data [14]. Finally, gene annotation, RNA-seq sequencing evaluation, gene-structure analysis, expression-level analysis, expression-variation analysis, and gene-enrichment analysis were carried out [15].

### 2.3. Quantitative PCR Assay

The total RNA of *U. prolifera* was extracted from each group followed by reverse-transcription into cDNA using the Fast-King cDNA first strand synthesis kit (Tiangen, Beijing, China). Then nine genes selected from the transcriptome were applied for qRT-PCR, where 18S rRNA was taken as the internal reference [12]. The target gene primers were designed using NCBI database online tool “Primer-BLAST” (Table S1), and Tiangen’s Talent fluorescence quantitative detection kit (SYBR Green) was used for the qPCR experiment, with the formulate as follows: 2 × Talent qPCR PreMix 12.5 μL, positive and negative primers 0.75 μL, cDNA template 1 μL, RNAase-free ddH_2_O 10μL. The reaction system was placed in the FTC-2000 PCR instrument, with the program setting as follows: 3 min pre-denaturation at 95 °C, 40 times of recycles including 95 °C for 30 s, 60 °C for 30 s, and 72 °C for 30 s. All samples had four repeats, and gene differential expression was calculated by 2^−ΔΔCT^ [16].

### 2.4. Proteomics Procedure

The samples were ground by liquid nitrogen and precipitated by TCA/acetone, then an appropriate amount of SDT lysate was added, respectively. The samples were bathed in boiling water for 15 min, then treated with ultrasonic treatment and centrifuged at 12,000× *g*. After the supernatant was collected, the protein was quantified by the BCA method [17], and the filtrate was collected by the FASP enzymatic hydrolysis method [18]. The peptides were desalted by the C18 Cartridge, then lyophilized and redissolved with 40 μL 0.1% formic acid solution. The peptides were quantified (OD_280_). High performance liquid chromatography was used to separate each sample using the HPLC liquid phase system easy NLC with nanositre flow rate. After chromatographic separation, Q-Exactive mass spectrometer (Thermo Fisher Scientific, Waltham, MA, USA) was used for mass spectrometry analysis. The mass charge ratio of polypeptides and fragments was collected as follows: after each full scan, 20 fragments were collected (MS2 scan). MS2 activation type was HCD, isolation window was 2 m/z, secondary mass spectral resolution was 17,500 at 200 m/z, normalized collision energy was 30 eV, and underfill was 0.1%.

### 2.5. Metabolomics Procedure

The sample was quantitatively weighed for liquid nitrogen grinding, dissolved in methanol acetonitrile aqueous solution (*v*/*v*, 2:2:1), centrifuged at 14,000× *g* at 4 °C for 20 min, and then the supernatant was taken. The supernatant was then redissolved in acetonitrile aqueous solution (acetonitrile: water =1:1, *v*/*v*) for mass spectrometry. The supernatant was taken for sample analysis after centrifugation at 14,000× *g* at 4 °C for 15 min. The samples were separated on an Agilent 1290 Infinity LC ultra-performance liquid chromatography (UHPLC) HILIC column. The samples were separated by UHPLC and analyzed by Triple TOF 6600 mass spectrometers (AB SCIEX, Boston, MA, USA). The obtained original data were converted into the MZML format by Proteo Wizard (Palo Alto, CA, USA), and then the XCMS program was used for peak alignment, retention time correction, and peak area extraction. Accurate mass number matching (<25 PPM) and secondary spectral matching were used for metabolite structure identification, and a database built by the laboratory was retrieved. The integrity of the data extracted by XCMS was checked. The metabolites with missing values of more than 50% in the group were removed and did participate in the subsequent analysis. The extreme values were deleted, and the total peak area was normalized for the data to ensure the parallelism of comparison between the samples and metabolites. After being processed, the data were input into the software SIMCA-P 14.1 (Umetrics, Umea, Sweden) for pattern recognition. After the data were preprocessed by pareto-scaling, multi-dimensional statistical analysis was conducted, including unsupervised principal component analysis (PCA), partial least squares discriminant (PLS-DA) and orthogonal partial least-squares discriminant (OPLS-DA) analysis. One-dimensional statistical analysis included student’s *t*-test and multiple of variation analysis, and volcano maps were drawn by R software (R Foundation for Statistical Computing, Vienna, Austria).

### 2.6. Lipidomics Procedure

After centrifugation at low temperature and high speed, the upper organic phase was taken, and the ammonia gas was blown dry. Isopropanol solution was added for resolution during mass spectrometry analysis. The samples were centrifuged for 15 min at 14,000× *g* under 10 °C in the vortex, and the supernatant was taken for sample analysis. The samples were separated by Nexera UHPLC LC-30A ultra performance liquid chromatography (Shimadzu Technologies, Kyoto, Japan). Electrospray ionization (ESI) positive and negative ion modes were used for detection, respectively. The samples were separated by UHPLC and analyzed by mass spectrometry with Q exactive plus mass spectrometer (Thermo Scientific, New York, NY, USA). Peak and lipid identification (secondary identification), peak extraction, peak alignment, and quantification were performed by lipaid search software version 4.1 (Thermo Scientific, New York, NY, USA). In the extracted data, lipid molecules of RSD > 30% were deleted. For the data extracted by lipaid search, lipid molecules with missing values > 50% in the group were deleted, and the total peak area was normalized for the data. SimCA-P 14.1 (Umetics, Umea, Sweden) was used for pattern recognition. After the data were preprocessed by Pareto-scaling, multi-dimensional statistical analysis was conducted, including unsupervised PCA, PLS-DA, and OPLS-DA analysis. One-dimensional statistical analysis included student’s *t*-test and multiple of variation analysis, and R software drew volcano maps, hierarchical clustering analysis, and correlation analysis.

## 3. Results

### 3.1. Basic Data of Transcriptome Analysis

Trimmomatic processed the raw data obtained by high-throughput sequencing to obtain the clean data. The average read length of each sample was more than 142 bp, with the total read length more than 39 Mb, the base amount more than 5.5 Gb, the GC ratio greater than 59%, and the Q30 ratio between 96.29 and 96.48%. It indicated good sequencing quality (Table S2). Trinity was used to assemble the clean data into transcripts with denovo assembly and remove redundancy. By taking the longest transcript in each transcript cluster as the unigene, 28,362 unigenes were obtained, with an average length of 1406 bp., wherein the longest sequence length was 26,903 bp (Table S3). After comparison, 1579 unigenes sequences were annotated in the databases of NR, KEGG, Swiss-prot, and KOG, and the numbers of annotated genes were 8502, 1801, 7670, and 5851, respectively (Figure S1). Compared with the control group, there were 100 genes whose expression quantities were extremely significantly up-regulated (|Log_2_Fold Change (FC)| > 2, and *p*-value < 0.05), and 167 genes were down-regulated for *U. prolifera* under high light intensity.

### 3.2. Target Gene Verification Results

**Figure 1:**
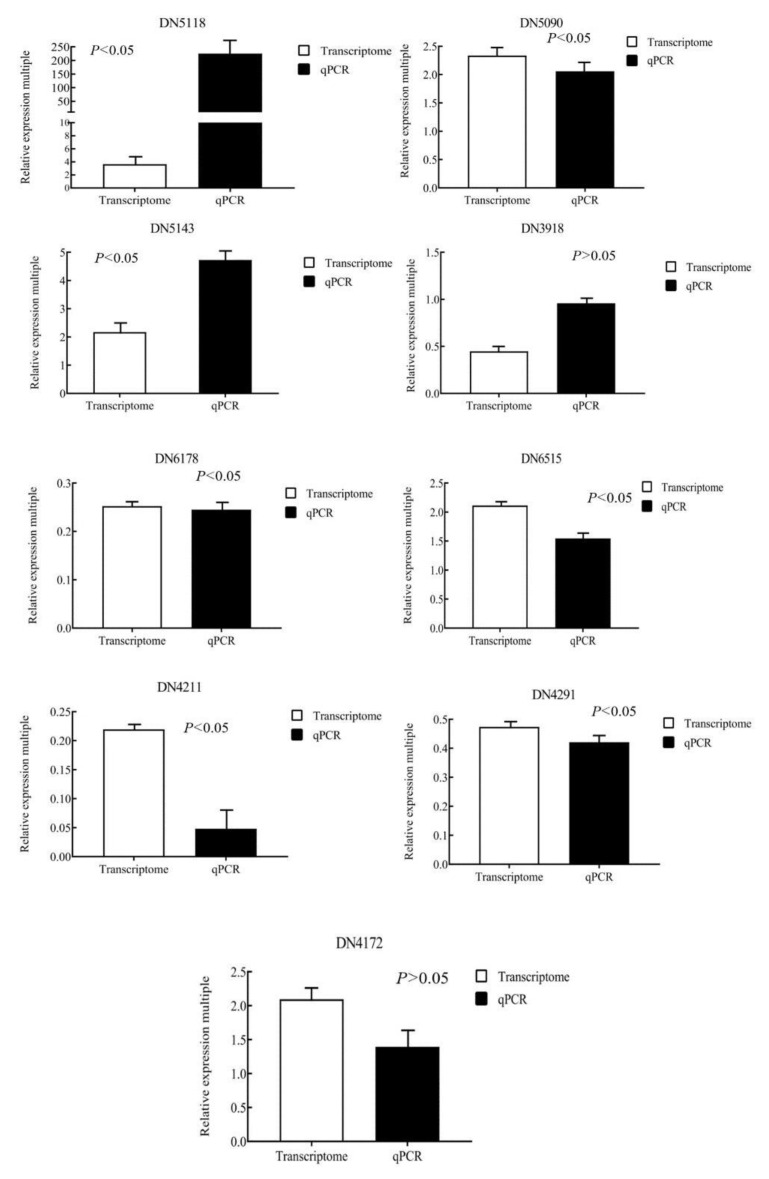
The nine significantly differential expressed genes in *U. prolifera* under high light intensity (400 μmol·m^−2^·s^−1^) were verified by qRT-PCR.

The selected nine genes were verified by real-time fluorescence quantitative PCR, and the data were analyzed. As shown in Figure 1, under the condition of 12 h high light intensity, the expression trends of nine genes were similar with the transcriptomics results, indicating that the transcriptome data were relatively reliable.

### 3.3. Basic Data of Proteome Analysis

According to the obtained mass spectrum, the Andromeda engine integrated by Max Quant was used for identification. The filtering was completed with PSM-level FDR ≤ 1%, and filtering was performed with protein-level FDR ≤ 1%. There were 18,100 identified peptide fragments and 2226 identified proteins. The unique peptide fragment is the protein’s characteristic sequence. In this experiment, there were 309 unique peptide segments with a quantity of two (Figure S2). The obtained proteins were mostly distributed between 10–50 kDa, of which 20–30 kDa had the most distribution (Figure S3). Max Quant was used for the quantitative analysis of each group with Welch’s *t*-test. It showed that the two groups contained 62 different proteins (|FC| ≥ 1.5, and *p* < 0.05), of which 21 were up-regulated, and 41 were down-regulated.

### 3.4. Basic Data of Metabolome Analysis

The chromatographic peak’s response intensity and retention time in the positive and negative ion mode of the QC samples in the metabolome overlapped. SIMCA-P software was used for PCA analysis to demonstrate that the parallel samples of each group were closely clustered together, which showed that the experiment had good repeatability. In the positive ion mode, 3790 ion peaks were obtained; in the negative ion mode, 3606 ion peaks were obtained. PLS-DA measured the strength of influence and interpretation of metabolites’ expression patterns on the classification of samples in each group by calculating variable importance for the projection (VIP). The PLS-DA model’s evaluation parameters R_2_Y = 0.997 (for positive ions) and 0.971 (for negative ions) after seven interactive verification cycles. OPLS-DA was modified based on PLS-DA to filter out noises unrelated to classified information, which improved the model’s analysis and effectiveness. In this model, R_2_Y = 1 (for positive ions) and 0.999 (for negative ions). In the above two models, R_2_Y was close to one, which explained the samples’ metabolic differences in the two groups. On this basis, 29 significantly different metabolites (VIP > 1 and *p* < 0.05) were identified through statistical analysis and screening, wherein 24 were up-regulated, and 5 were down-regulated.

### 3.5. Basic Data of Lipidome Analysis

The response intensity and retention time in the UHPLC-Obitrap MS BPC of QC samples in the lipidome showed that the experiment had good repeatability. The PLS-DA and OPLS-DA models’ evaluation parameters R_2_Y were equal to 0.968 and 0.993, respectively, which explained the metabolic differences between the samples in the two groups. In this study, 558 lipid molecules were identified with 21 subclasses, mainly involving triglyceride (TG), ceramidesglycerol 1 (CerG1), diacylglycerol (DG), DGDG, diacylglycerol monoacylglycerol (DGMG), MGDG, monogalactosyl monoacylglycerol (MGMG), phosphatidylglycerol (PG), and sulfoquinovosyl diacylglycerol (SQDG) (Figure S4). There were five significantly different metabolites (VIP > 1, and *p* < 0.05), among which one was up-regulated, and four were down-regulated (Table S4).

Lipid group results showed 76 DGDG expressions, with OPLS-DA model VIP > 1 as the standard. In the high light experimental group, 13 DGDG were differentially expressed, and 7 were up-regulated. Among them, DGDG ((16:4/18:4) + HCOO) was up-regulated extremely significantly (*p* <0.01, and FC = 3), and the obtained DGDG had the longest carbon chain and the lowest saturation. The work detected a total of 53 MGDG (the direct precursor of DGDG biosynthesis), where 11 differential expressions were down-regulated, including the products (MGDG (16:4/18:4) + HCOO) corresponding to the above-mentioned DGDG. Among them, the difference of MGDG ((16:0/16:4) + HCOO) was extremely significant (*p* = 0.013, and FC = 0.69), and the product was supposed to be a reaction intermediate. The work detected twenty monogalactosyl monoacylglycerols (MGMGs), of which seven were differentially expressed, and five were down-regulated. MGMG ((16:1) + HCOO, FC = 0.60) and MGMG ((16:2) + HCOO, FC = 0.72) had extremely significant difference (*p* < 0.05), with intermediate saturation. Correlation analysis showed that these two MGMGs were closely positively correlated with the expression of MGDG and had a strong negative correlation with DGDG. Besides, 42 DAGs, 16 PGs, 6PAs, and 6 fatty acids (including palmitic acid and cis-9-octadecenoyl-CoA) were detected in the lipid group, but no differential expression was found. Six PIs and seven PEs detected were not different, indicating that the above intermediate products did not participate in the endoplasmic reticulum reaction.

### 3.6. Photosynthesis of U. prolifera in the Conditions of High Light Stress

Through multi-omics joint analysis, it was found that some important genes related to the process of photosynthesis in *U. prolifera* changed significantly after 12 h of high light intensity (Table 1). Transcriptomics data showed that genes that promote chlorophyll and carotenoid synthesis were up-regulated, e.g., glutamate-1-semialdehyde 2,1-aminomutase, ABC transporter C family member 3, and photosystem II CP43 reaction center protein. On the other hand, the expression of pheophytinase decreased. The genes involved in promoting the synthesis of photosystem I and electron transport were up-regulated, e.g., photosystem I P700 chlorophyll apoprotein A1, cytochrome b6-f complex subunit 4, and ATP synthase subunit. Meanwhile, the PSII complex-related gene expression was down-regulated, e.g., photosystem II protein D2 (psbD) and aldedh domain-containing protein. The gene expression associated with the dark reaction process in photosynthesis, e.g., carbonic anhydrase was down-regulated. The expression of genes associated with phosphoenolpyruvate synthesis was up-regulated and metabolism was down-regulated, e.g., pyruvate, phosphate dikinase, phosphoenolpyruvate/phosphate translocator 1 and phosphoenolpyruvate carboxylase 1.

Proteomics data showed that the expression of proteins related to the biosynthesis of chlorophyll was up-regulated, e.g., uroporphyrinogen, heat shock protein 90-5, chloroplastic, and ABC transporter C family member 2 and the expression of C3 pathway-related proteins was inhibited, e.g., pyridoxal 5’-phosphate synthase subunit PDX1.

The combined metabolome and lipidome data showed that the content of photosynthetic membrane involved in photosynthesis increased, e.g., DGDG. However, MGDG as an intermediate for DGDG production decreased, indicating that 12-h high light stress promoted the synthesis of the photosynthetic membranes of *U. prolifera*. In summary, 12 h of intense high light stress promoted the synthesis of chlorophyll and carotenoid, PSI, and electron transport subunit, and complemented ATP deficiency by coupling with cyclic electron transport. Meanwhile, it weakened PSII synthesis and acyclic photophosphorylation, reduced NADPH generation, and inhibited carbohydrate synthesis in a dark reaction. Meanwhile, a shift from the C3 to the C4 pathway started by the promotion of phosphoenolpyruvate synthesis, while inhibiting phosphoenolpyruvate transport and consumption. Furthermore, high light induced a large amount of DGDG synthesis on the photosynthetic membrane while consuming the substrate MGDG. Those might be supplemented by MGMG. It was suggested that the 12 h time point was the turning point of *U. prolifera* tolerant to high light.

### 3.7. Energy Metabolism of U. prolifera in Conditions of High Light Stress

Through multi-omics joint analysis, it was found that after 12 h of intense light stress, some important genes related to energy metabolism in *U.prolifera* changed significantly (Table 2). Transcriptomics data indicated that the expression of genes related to energy metabolism was down-regulated, such as adenylate kinase 5, acyl-CoA-binding domain-containing protein 5, and pyruvate dehydrogenase E1 component subunit α-1. The expression of genes related to redox activity was also down-regulated, e.g., protein tas, cytochrome P450 4e3, and amino oxidase domain-containing. On the other hand, the transcriptome showed that the expression of genes that are involved in glycolysis was up-regulated, e.g., endoglucanase E-4 and endoglucanase 1 and both enzymes catalyzing the endohydrolysis of 1, 4-β-glucosidic linkages in cellulose, lichenin, and cereal β-D-glucans. Meanwhile, the gene expression of 4-α-glucanotransferase DPE2, which catalyzes starch to sucrose, was also up-regulated.

The proteome showed that the expression of proteins related to energy metabolism was up-regulated, e.g., R-mandelonitrile lyase-like, NADH: ubiquinone oxidoreductase 30 kDa.

The metabolome showed that the content of metabolites related to TCA increased, e.g., L-malic acid, L-asparagine, and cyclohexylamine. Meanwhile, that of diethanolamine and ribitolalso increased. However, the content of sucrose decreased.

As a whole, it was found that after short-term high light stress, sucrose content and glycolysis gradually increased, while the TCA cycle gradually weakened, including the reduction in acetyl-CoA production and transport and reduction in proton production, respiratory terminal oxidase production, GTPase synthesis, and ATP production. Thus, the overall trend of energy metabolism was down-regulated which might make *U. prolifera* dormant.

### 3.8. Transcription and Translation of U. prolifera in Conditions of High Light Stress

Through multi-omics joint analysis, it was found that some important genes related to the process of protein synthesis and expression in *Ulva* changed significantly after 12 h of high light intensity (Table 3). Transcriptomics data showed that genes that activate DNA were down-regulated, e.g., DOT1 domain-containing protein, ATP-dependent DNA helicase DDM1, and RuvB-like 2; as well as those in RNA transcription, e.g., AP2-like ethylene-responsive transcription factor AIL5, transcriptional activator Myb, ESF1 homolog, and transcription initiation factor TFIID subunit 5. However, genes involved in exosome-mediated RNA decay were up-regulated, e.g., tetratricopeptide repeat protein SKI3. Furthermore, genes related to protein synthesis and degradation were down-regulated, e.g., ribosome biogenesis protein BRX1 homolog, tRNA pseudouridine synthase A, and general transcription factor 3C polypeptide 5. However, Carboxypeptidase inhibitor SmCI involved in the inhibition of pancreatic carboxypeptidase was up-regulated. Nucleotide synthesis was inhibited, e.g., 5’-nucleotidase, pseudouridine-5’-phosphate glycosidase, and cytosolic purine 5’-nucleotidase.

Proteomics data showed that the expression of proteins related to protein synthesis was down-regulated, e.g., pre-mRNA-splicing factor ATP-dependent RNA helicase DEAH3, eukaryotic translation initiation factor 5A-2, and DNA-binding helix-turn helix protein. The combined metabolome data showed that the content of some amino acid increased, e.g., L-glutamate, L-methionine, and L-glutamate.

In summary, after 12 h of intense light stress, the expression of protein synthesis-related genes showed an overall trend of down-regulation in *Ulva* algae, including DNA activation, RNA transcription, protein synthesis, and degradation. Meanwhile, it also inhibited nucleotide production. This showed that 12 h of high light stress is the turning point of *U. prolifera* tolerant to the condition of high light stress.

### 3.9. Signal Transduction, Ion Transport, and Cytoskeleton Synthesis of U. prolifera in Conditions of High Light Stress

According to omics data, some important genes related to signal transduction and growth altered significantly after 12 h of high light stress (Table 4). Transcriptomics data indicated that the expression of genes related to signal transduction were down-regulated, e.g., adenylate cyclase and protein RRC1. Moreover, ABC transporter G family member 31 that suppresses radicle extension and subsequent embryonic growth was up-regulated, while GPCR-type G protein 2 that is required for seedling growth and fertility was down-regulated. On the other side, the expression of genes related to ion transport was also down-regulated, e.g., potassium/sodium hyperpolarization-activated cyclic nucleotide-gated channel 2, sodium/calcium exchanger 3, and sodium- and chloride-dependent GABA transporter 2. Many genes on cytoskeleton synthesis were down-regulated, e.g., kinesin-like protein KIN-4A, tubulin glycylase 3A, and protein tilB homolog, while several of them were up-regulated, e.g., tubulin polyglutamylase TTLL4, dynein assembly factor 5, and flagellar associated protein.

Proteomics data showed that the expression of proteins involved in signal transduction was down-regulated, e.g., inositol, and calcium-dependent protein kinase 22. The expression of related proteins mediating mitochondrial protein transport was down-regulated, e.g., mitochondrial import inner membrane translocase subunit Tim9. However, UPF0187 protein At3g61320 which participates in the formation of anion channels was up-regulated, as was the transmembrane transport proteins, e.g., vesicle-fusing ATPase and ATP-energized ABC transporter.

Metabolomics data showed that a few metabolites involved in signal transduction were up-regulated, such as L-glutamate, adenosine, and succinate, while isoleucyl-glutamate was down-regulated.

In summary, after 12 h of high light stress, *U. prolifera* showed a decrease in signal transduction generation, inhibition of growth-related gene expression, weakened ion transport and microfibril and microtubule synthesis, and overall inhibition of cilium synthesis. Therefore, cytoskeleton synthesis was generally inhibited, and cell growth was limited.

### 3.10. Cell Division, Gametogenesis, and Apoptosis of U. prolifera in Conditions of High Light Stress

According to multiple omics analysis, it was found that after 12 h of high light stress on *U. prolifera*, some important genes in the process of cell division, gametogenesis, and apoptosis in *U. prolifera* had significant changes (Table 5). Transcriptomics data indicated that the expression of many genes involved in cell division was down-regulated, e.g., DNA mismatch repair protein MSH4, DNA replication licensing factor MCM5, single mybhistone 3, and heat shock-like 85 kDa, and some genes promoting the cell division were up-regulated, e.g., histone acetyltransferase MCC1, protein chromatin remodeling 24, and serine/threonine-protein kinase mos.

Transcriptomics data indicated that the expression of genes involved in gametogenesis were down-regulated, e.g., thioredoxin domain-containing protein 3 homolog, 26 S proteasome non-ATPase regulatory subunit 12 homolog, and cilia- and flagella-associated protein 91, while C-factor which is necessary for spore differentiation was up-regulated. Moreover, some genes are expressed to inhibit apoptosis, e.g., serine/threonine-protein kinase, dnaJ homolog subfamily A member 1, and WD repeat-containing protein 35. However, some genes are expressed to promote apoptosis, e.g., metacaspase-1.

Proteomics data showed that the expression of proteins that promote cell division was down-regulated, e.g., SNF1-related protein kinase regulatory subunit gamma and R-mandelonitrile lyase, while dnaJ protein homolog 2 which plays a continuous role in plant development was up-regulated.

According to the omics data above, it was found that after 12 h of high light, the gene expression related to cell division and gametogenesis showed an overall downward trend in *U. prolifera*. At the same time, the expression of apoptosis-related genes changed, which means the reproductive development of *U. prolifera* was inhibited by high light stress conditions. It was speculated that 12 h of high light intensity is the turning point of *U. prolfera* cell division and reproduction.

### 3.11. Resistance of U. prolifera in Conditions of High Light Stress

According to multiple omics analysis, it was found that after 12 h of high light stress on *U. prolifera*, some important genes related to resistance in *U. prolifera* had significant changes (Table 6). The transcriptome showed that many genes were expressed to increase resistance to stress. Some were involved in disease, e.g., disease resistance protein RGA4, disease resistance protein TAO1, and TMV resistance protein N; some were involved in extradition, e.g., Broad substrate specificity ATP-binding cassette transporter ABCG2 and neurotrypsin. Others were related to salt tolerance, e.g., mannitol dehydrogenase; DNA damage tolerance, e.g., disease resistance protein RPP5. However, a few genes were expressed to decrease resistance. They were related to cellular defense responses, e.g., DEAD-box ATP-dependent RNA helicase 50, 17.6 kDa class I heat shock protein 3, and activator of 90 kDa heat shock protein ATPase homolog 1; or DNA damage repair, e.g., deoxy ribodipyrimidine photo-lyase; or photoprotection, e.g., carotene biosynthesis-related protein CBR (other specific categories are shown in Table 6).

Proteomics data showed that the expression of proteins involved in antioxidant response was down-regulated, e.g., glutathione S-transferase, ascorbate peroxidase, and peroxidase, as was glutamate-cysteine ligase which is involved in detoxification. However, 10 kDa chaperonin which promotes the refolding and proper assembly of unfolded polypeptides generated under stress conditions was up-regulated.

According to the metabolome data, most of the metabolites on anti-stress were up-regulated, including γ-L-glutamyl-L-glutamic acid, L-glutamate, and D-proline. However, there were also metabolites that were down-regulated, e.g., galactinol, L-pyroglutamic acid, and L-glutamine.

According to omics data above, it was found that after 12 h of high light stress, some aspects of resistance were enhanced, including disease, extrudation, salt tolerance, DNA damage tolerance, stress adaptation, lipoxygenases activity, riboflavin, degradation, calcium depend kinase activity, salicylic acid synthesis, and cell recognition and adhesion; others were down-regulated, including cellular defense responses, DNA damage repair, photoprotection, antioxidant and innate immunity. It seemed that in various ways, *U. prolifera* employ mechanisms to cope with light stress conditions.

### 3.12. Cell Membrane Synthesis and Repair of U. prolifera in Conditions of High Light Stress

According to the omics data, some important genes related to cell membrane synthesis and repair altered significantly after 12 h of high light stress (Table 7). Transcriptomics data indicated that the expression of genes related to cell membrane, cytoderm, and plasmodesmata synthesis was up-regulated, while lipid alienation was down-regulated, e.g., Enoyl-[acyl-carrier-protein] reductase [NADH], sterol sensor 5-transmembrane proteinand UDP-glucuronate 4-epimerase 1. Moreover, genes associated with tRNA synthesis were up-regulated, e.g., tRNA 2’-phosphotransferase and ribonuclease Z, mitochondrial.

Proteomics data showed that the expression of proteins involved in lipid biosynthesis was up-regulated, e.g., CDP-diacylglycerol-serine O-phosphatidyl transferase 2 and adipocyte plasma membrane-associated protein.

The combined metabolome and lipidome data showed that the content of certain metabolites increased during the process of fatty acid biosynthesis, e.g., cis-9-palmitoleic acid, myristic acid, and palmitic acid. There were also decreased ones, e.g., 4,7,10,13,16,19-docosahexaenoic acid in metabolome, and MGDG (16:0/16:4) + HCOO and MGMG (16:1) + HCOO in lipidome.

In summary, after 12 h of intense light stress, genes were expressed to promote the formation of the cell membrane, cytoderm, and plasmodesmata, and reduce lipid dissimilation metabolism. Moreover, synthesis of tRNA was also promoted, which might be related to promotion cell repair.

## 4. Discussion

### 4.1. High Light Intensity Conditions Affecting the Composition of Photosynthetic Membranes

When the external environment changes drastically, algae have evolved multiple mechanisms to avoid harm [19,20]. The algae cell membrane is a significant hydrophobic barrier separating it from the surrounding environment [21]. Therefore, maintaining or regulating the physical and biochemical properties of cell membranes is very important. Regarding the thylakoid-membrane glycerolipids for photosynthesis and photoprotection in chloroplasts, different light conditions will affect them [22,23]. Unsaturated fatty acids are also important components of biofilms [24]. They can increase the fluidity of the membrane, which is important for activating the enzymes on the membrane [25,26].This transcriptomics data (Table 7) showed that the transcript expressions of the sterol sensor 5-transmembrane proteins involved in sterol synthesis were up-regulated. Sterols are essential eukaryotic lipids that are required for a variety of physiological roles [27]. Under the condition of high light, the photosynthetic membrane of *U. prolifera* was damaged to a certain extent, which accelerated the repair process to ensure the normal photosynthesis of the body [28].

Metabolomics data (Table 7) showed that metabolite 4,7,10,13,16,19-docosahexaenoic acids involved in the synthesis of unsaturated fatty acids decreased; while α-linolenic acid and linoleic acid were up-regulated. The biosynthesis of these fatty acids was corelated because linoleic acid was the synthetic precursor of α-linolenic acid, and the latter was the synthetic precursor of docosahexaenoic acid. The product of fatty acid metabolism was the precursor of lipoic acid metabolism, which involved ferredoxin-thioredoxin reductase. The thioredoxin has been recognized as the key system for transmitting the light-induced reduction signal to the target proteins [29,30].

DGDG and MGDG are the main membrane lipids (Table 7) that constitute the chloroplast photosynthetic membrane of higher plants, accounting for more than 80% of the chloroplast membrane lipids [31]. Among them, DGDG is one of the most important compounds constituting photosynthetic membranes and exists in almost all biofilms [32]. It accounts for more than 20% of total lipids and can replace other phospholipids under special circumstances [33]. Moreover, DGDG plays an important role in maintaining the oligomer structure of the photosystem II light-harvesting pigment–protein complex and regulating the photosystem II and the oxygen-evolution activity of its core complex [34,35]. The metabolomics results of this study showed that 12-h high light stress increased DGDG content and decreased MGDG content at the same time. In higher plants containing a large quantity of hexadecenoic acids, the biosynthesis of DGDG started from palmitic acid and became cis-9-octadecenoyl-CoA through acetylation, chain lengthening, and hydrogenation [36]. The latter reacted with 3-phosphoglycerol to form lysophosphatidic acid [37] and then deacylated to form phosphatidic acid. Phosphatidic acid as a substrate could generate phosphatidylglycerol and DAG. DAG reacted with uridine diphosphate galactose to generate MGDG under the catalysis of MGDG synthase. The latter was combined with galactose-1-phosphate and finally generated DGDG under the catalysis of DGDG synthase [38]. In summary, it was speculated that high light induced a large amount of DGDG synthesis on the photosynthetic membranes and consumed the substrate MGDG, which could be supplemented by MGMG [39].

### 4.2. Changes in Photosynthetic Pigments Affected by High Light Stress Conditions

The content of chlorophyll can be induced by light. In this study, the expression of chlorophyll and carotenoid-related genes (Table 1) was enhanced under 12 h high light stress (400 μmol·m^−2^·s^−1^). The *Chl* a and yield of *U. prolifera* cultured under weak light (62 μmol·m^−2^·s^−1^) for one day were twice that under high light conditions (324 μmol·m^−2^·s^−1^). However, within one week of culture, there was no difference in the *Chl* a yield of all samples [40]. The synthesis of *Chl* a in the red alga *Corallina elongata* can be induced by red light pulses [41] and regulated by light intensity. After 5 h of light treatment, the pigment reached a steady state. When the irradiance increased, chlorophyll synthesis also increased, indicating that this steady state was dynamic [42]. For floating *U. prolifera*, the surface and lower layers of the algal mat had different photosynthetic responses [43]. The surface algae mat dissipated excess energy through the quantum control of photosynthesis (energy quenching or redistribution between PSⅡ/PSI) and reduced the photosynthetic system’s damage. The lower algal mat increased *Chl* a and *Chl* b and reduced the ratio of *Chl* a/b to improve its ability to use light energy [44]. Therefore, *U. prolifera* has strong photosynthetic plasticity [45,46]. Due to the waves’ interference, it quickly adapted to the frequent exchange between the surface and the lower environments through the change of pigment compositions, energy quenching, and energy redistribution between PSⅡ/PSI [44].

Chlorophyll synthesis and catabolism were dynamically balanced, and the change in the ratio of *Chls* a/b under different physiological conditions was reflected in this experiment’s transcriptome data. After 12 h of high light treatment, the expressions of glutamate-1-semialdehyde 2,1-aminomutase-related genes (Table 1) were up-regulated to promote the production of *Chl* b [47,48]. The expression level of pheophorbide hydrolase-related genes, which was the key rate-limiting enzyme of the chlorophyll catabolism pathway, was down-regulated. It slowed down the communication of *Chl* b to *Chl* a, and reduced the ratio of *Chl* a/b. However, *Chl* a increased, thereby improving its ability to use light energy as a whole. The contents of the photosystem’s auxiliary proteins and pigments were regulated by light. Under 700 μmol·m^−2^·s^−1^, the chlorophyll content in *Ulva* sp. decreased within a few minutes, while the carotenoids remained unchanged [49]. Moreover, under 800 μmol·m^−2^·s^−1^, the upper layer of the meadow was involved in the gene up-regulation of light adaptation (rubisco, ferredoxin, and chlorophyll-binding protein) and light protection (antioxidant enzymes, genes related to lutein cycle, and tocopherol biosynthesis), indicating the activation of more defense mechanisms [50]. However, under the condition of 400 μmol·m^−2^·s^−1^ for 12 h, the antioxidant ability of *U. prolifera* decreased, but the expression of disease resistance, DNA tolerance, and other defense mechanisms was enhanced (Table 6). In addition to common photosensitive pigments such as carotenoids and chlorophyll, seven rhodopsin types, two leuco dyes, and one photoprotein were found in *Chlamydomonas reinhardtii*, wherein rhodopsin was a flavin-based photoreceptor sensitive to blue light [51]. Among the light-harvesting proteins, LHCX and LHCZ genes had a stronger up-regulation effect under 400 μmol·m^−2^·s^−1^ than that under 60 μmol·m^−2^·s^−1^ [52]. The similar proteins ElipL1, ElipL2, Cbrx, and OHP in *U. linza* were also upregulated by high light within 3 h under 2000 μmol·m^−2^·s^−1^ [53]. However, photolyase (Table 6), a blue-light receptor that could bind to folic acid and FAD, was down-regulated in this study, which showed that the blue light sensitivity of *U. prolifera* weakened after 12 h of high light. Photolyase can repair UV-induced DNA damage in a light-dependent manner and the plant’s blue light photoreceptors, which mediate light-dependent regulation of seedling development [54], e.g., the germination of *U. prolifera* spores [55]. Moreover, blue light can improve the photosynthetic rate of *Ulva* sp. [56]. Thus, the rate of photosynthesis of *U. prolifera* decreased because its sensitivity to blue light was weakened. However, the study showed that 12 h of high light stress could promote the expression of DNA repair process of *U. prolifera* (Table 6). Therefore, it meant that *U. prolifera* actively reduced photosynthetic rate and growth rate in response to 12-h high light stress under the premise of protecting DNA.

*U. prolifera* has a mechanism to resist photoinhibition. When the required light energy exceeds the range that the photosynthetic system can withstand, the photosynthetic function declines and light inhibition occurs. Plants have multiple protection mechanisms in response to excessive light energy [57]. For example, *Macrocystis pyrifera*, *Chondrus crispus*, and *Ulva lactuca* promote self-shading by increasing biomass and reduce photoinhibition [58]. The dinoflagellate uses the flavin cycle for photoprotection through heat dissipation [59]. When the light intensity exceeded 400 μmol·m^−2^·s^−1^, the electron flow reached saturation, with the increased excitation pressure and NPQ [60]. When *Ulva fasciata* was exposed to 1500 μmol·m^−2^·s^−1^, protein D1 rapidly degraded, and its PES medium form was destroyed. The NPQ ability decreased to a steady state within 110 min, but it quickly recovered in low light [61]. When non-photochemical quenching did not work properly, *U. fasciata* maintained NPQ by keeping a small proportion of high fast-light PSⅡ combinations. However, there was high-thermal activity with high light because the degradation of Cyt_6_f seriously hindered electrons’ transmission, which led to NPQ [62]. The results of this study showed that *U. prolifera* attenuated PSII synthesis and acyclic photosynthetic phosphorylation, and enhanced respiration, and the carbon fixation mode changed from C3 pathway to C4 pathway. At the same time, RRC1 which is required for phytochrome B (phyB) signal transduction [63] was reduced and phyB is a major photoreceptor in plants [64].

Far-red light enhanced the circulating electron flow around PSI and induced the expression of LHCSR to trigger NPQ in *U. prolifera*. Lhcb1 and CP29 were adjusted up-regulated under FRL, which meant that the PSⅡ antenna size increased [65]. NPQ induction could be related to individual proteins, for example, *psb*S content was positively correlated. The latter played an important role in reconstructing the PSII-CLHCII super complex and the energy balance regulation of the thylakoid membrane [66], Or it was regulated by zeaxanthin and triggered and controlled by the transthylakoid proton gradient (ΔpH) under high light (1954 μmol·m^−2^·s^−1^). More importantly, it was regulated by the assemblage of the light-harvesting complex (LHC) family. Under high light conditions, the expression of LHCSR was even higher than that of PSBS [67]. However, *U. prolifera*’s NPQ lacked a rapid activation mechanism under high light, and its monomeric LHC proteins only contained CP29 and CP26 instead of CP24. Furthermore, a significant increase in the expression level of CP26 did not change the concentrations of the photoprotective proteins psbS and lhcSR, with the gradual synthesis of zeaxanthin. The atypical NPQ made *U. prolifera* more suitable for the complex sea environment [62]. The transcriptome data in this experiment also showed that under high light (400 μmol·m^−2^·s^−1^) treatment for 12 h, *U. prolifera* was involved in light capture, PSI and PSII, and the expressions of genes related to photosynthetic pigment synthesis (Table 1). It was very similar to the atypical NPQ of *U. prolifera*.

### 4.3. High Light Affecting the Signal Transduction Pathways of U. prolifera

Light-induced cAMP changes significantly increase stress-response proteins in *Arabidopsis*, so adenylate cyclase may act as a light sensor in higher plants [68]. The transcript expression quantity of *cya*C, the primary subtype of adenylate cyclase in algae, is strongly affected by light, which is about 300 times stronger than that of the dark-treated control group [69]. Under subsaturated white light irradiation, the oxygen release of photosynthesis is correlated with cAMP change, showing that electron transfer can regulate the accumulation of cAMP in *G. sesquipedale* and *U. rigida*, that is, cAMP level is regulated by light intensity [70]. The transcriptome data here showed that the transcript expression of adenylate cyclase was down-regulated (Table 4). At the same time, metabolome data showed that the expression of related metabolites involved in signal channels was up-regulated (Table 4).

Light can change calcium-dependent protein kinases. In plants, the multi-gene family of CDPKs (calcium-dependent protein kinase) encodes structurally conserved single-molecule calcium sensor/protein kinase, which plays essential roles in multiple signal transduction pathways. In this experiment, after 12 h of high-light stress for *U. prolifera*, the protein content of CDPKs was significantly down-regulated (Table 4), but the expressions of CDPKs-related transcripts were significantly up-regulated, indicating a large consumption of CDPKs and an increased demand. However, the transcript expressions of Calcium: Cation Antiporter, glucose 6-phosphoric acids/phosphates, and phosphoenolpyruvate/phosphate anti-transporter proteins, and K^+^-channel ERG-related proteins (including PAS/PAC sensor domain and K^+^-channel KCNQ) were significantly down-regulated (Table 4), indicating that the demands for calcium and potassium-ion transport inside and outside the membrane decreased. Besides, changes in light intensity promoted the release of cations to the outside of the cell quickly. For example, within the first two minutes, light caused the release of sodium ions in *Ulva lobata* and *Ulva expansa* to be twice that of the group in the dark, with ^86^Rb^+^ and ^85^Sr involved in the tracer released [71]. However, in addition to HCO^3−^, other anions such as ^36^Cl^−^, ^35^SO_4_^2−^ and [^14^C] acetate were not affected by light [72].

### 4.4. Growth and Stress-Resistance of U. prolifera under High Light

Light is an essential factor in controlling the growth of algae, and light intensity affects the algae’s growth and metabolism [73]. When the sunshine duration exceeds 12 h per day, compared with the light intensity of 10 μmol·m^−2^·s^−1^, the chloroplast surface area of *Ulva* spp. under 100 μmol·m^−2^·s^−1^ increases with the increasing light duration [74]. Under low light intensity, the long light period will also promote spores in *Ulva* spp. The algae’s growth rate in the light period (150 AE·m^−2^·s^−1^) is significantly higher than that in the dark period. It increases by about two times within 24 h because actin ACT expression is induced and inhibited by light and dark treatments [75]. However, in this experiment, under 400 μmol·m^−2^·s^−1^, the expression transcript of SDA1 involved in regulating the actin skeleton was down-regulated (Table 3), suggesting that cell growth was inhibited [76]. The enhancement of endopeptidase activity was considered the main reason for the decreased protein content during plant senescence [77], and the growth of *U. prolifera* under high light was also inhibited [78]. The up-regulated endopeptidase in this transcriptome data (Table 5) was also involved in the overall down-regulation of cell cycle checkpoint kinase expression of cyclin-dependent kinase CDK1 (the main engine of mitosis) and impaired cell cycle regulation.

The growth and distribution of algae are affected by many environmental factors such as temperature, light, and chemicals in the water. Relevant studies have gradually discovered algae stress-related proteins that resist adversity stress [79,80]. In this study, dnaJ protein. (Table 5), which was up-regulated in the proteome and down-regulated in the transcriptome, used as co-chaperone for HSP70 [81], and involved in stress resistance [82].

## 5. Conclusions

Based on four omics data analysis, high light stress mainly affected the mutual conversion of pentose and glucuronic acids, fatty acid biosynthesis, steroid biosynthesis, photosynthesis, pyrimidine metabolism, and carbohydrate metabolism, and other metabolic pathways, and regulated the cell cycle in *U. prolifera*. DGDG and MGDG metabolites were regulated by influencing the ascorbic acid and alginate metabolism, fatty acid metabolism, and energy metabolism to control changes in the photosynthetic membranes of *U. prolifera*, thereby affecting its photosynthesis. The results provide a further study on the mechanism of *U. prolifera*’s tolerance to high light stress and have laid a foundation for solving the reason of *U. prolifera* green tide.

## Figures and Tables

**Table 1:** Fluctuation of molecules in *U. prolifera* on photosynthesis after 12 h treatment with high light.

Function	Transcript ID	Annotation	Accession No.	FC ^a^
Chlorophyll and carotenoid synthesis	TRINITY_DN4172_c0_g2	Glutamate-1-semialdehyde 2,1-aminomutase	sp|Q39566|	2.11
	TRINITY_DN6224_c0_g1	ABC transporter C family member 3	sp|Q54JR2|	2.97
	TRINITY_DN4689_c0_g2	Photosystem II CP43 reaction center protein	sp|Q06J66|	2.60
	TRINITY_DN6515_c0_g5	Prolycopene isomerase	sp|Q8S4R4|	2.11
	TRINITY_DN6178_c1_g6	Pheophytinase	sp|Q9FFZ1|	0.25
Photosystem I and electron transport synthesis	TRINITY_DN5143_c0_g2	Photosystem I P700 chlorophyll apoprotein A1	sp|Q3ZJ07|	2.17
	TRINITY_DN15111_c0_g1	Cytochrome b6-f complex subunit 4	sp|Q3ZJ11|	3.07
	TRINITY_DN8870_c0_g1	ATP synthase subunit b	sp|Q3ZIZ8|	3.07
PSII complex Synthesis	TRINITY_DN4689_c0_g1	Photosystem II protein D2 (psbD)	tr|A0A0E3D7S4|	0.33
	TRINITY_DN6034_c0_g1	Aldedh domain-containing protein	tr|A0A059LN36|	0.42
Dark reaction process	TRINITY_DN6248_c1_g3	Carbonic anhydrase 5A	sp|P43165|	0.36
Phosphoenolpyruvate synthesis	TRINITY_DN5118_c0_g1	Pyruvate, phosphate dikinase	sp|Q42736|	3.63
	TRINITY_DN5977_c2_g1	Phosphoenolpyruvate/phosphate translocator 1	sp|Q69VR7|	0.33
	TRINITY_DN5718_c0_g1	Phosphoenolpyruvate carboxylase 1	sp|P81831|	0.46
Function	Protein ID	Annotation	Accession No.	FC ^b^
Chlorophyll synthesis	Maker04609	Uroporphyrinogen decarboxylase	sp|Q42855|	3.83
	Maker01827	Heat shock protein 90-5, chloroplastic	sp|Q9SIF2|	1.59
	Maker02125	s1-domain-containing protein	sp|Q93VC7|	1.74
	Maker05812	ABC transporter C family member 1	sp|Q9C8G9|	2.63
	Maker06519	ABC transporter C family member 2	sp|Q42093|	1.92
C3 pathway	Maker00363	pyridoxal 5′-phosphate synthase subunit PDX1	sp|Q9AT63|	2.04
Function	Description	Adduct	VIP	FC ^c^
Photosynthetic membrane synthesis	Palmitic acid	(M^−^H)^−^	2.81	2.11
Fuction	Lipid Iond	VIP	FC ^d^	
Photosynthetic membrane synthesis	DGDG (16:4/18:4) + HCOO	1.26	3.01	
	MGDG (16:0/16:4) + HCOO	1.19	0.69	

^a^ FC: 1 ≤ |Log_2_FC| < 2 indicated a significant difference of gene expression (q-value (modified *p* value after multiple hypothesis tests) < 0.05.), while |FC | ≥ 2 indicated extremely remarkable differences of gene expression. *n* = 3 biologically independent experiments. ^b^ FC ≥ 1.5 indicated that protein increased significantly (*p*-value < 0.05), while FC ≤ 0.6 indicated a significant decrease in protein. *n* = 3 biologically independent experiments. ^c^ FC > 1 indicated that protein increased, while FC < 1 indicated a decrease in protein. *p*-value < 0.05 means significant difference, *p*-value < 0.01 means extremely remarkable difference. *n* = 6 biologically independent experiments. “+” means cation of metabolite, while “-” means anion of metabolite. In all selected metabolites above, the VIP score > 1.0. ^d^ FC > 1 indicated that protein increased, while FC < 1 indicated a decrease in protein. *p*-value < 0.05 means significant difference, *p*-value < 0.01 means an extremely remarkable difference. *n* = 6 biologically independent experiments. In all selected lipids above, the VIP score > 1.0. The description of “a, b, c, d” in the follow tables is the same.

**Table 2:** Fluctuation of molecules in *U. prolifera* on energy metabolism after 12 h treatment with high light.

Function	Transcript ID	Annotation	Accession No.	FC ^a^
Energy metabolism	TRINITY_DN5770_c1_g1	Adenylate kinase 5	sp|Q8VYL1|	0.28
	TRINITY_DN5617_c0_g1	Acyl-CoA-binding domain-containing protein 5	sp|Q8RWD9|	0.46
	TRINITY_DN6220_c3_g3	Pyruvate dehydrogenase E1 component subunit α-1	sp|P52901|	0.46
	TRINITY_DN5251_c0_g1	Glycerol-3-phosphate dehydrogenase [NAD (+)] 1	sp|Q9SCX9|	0.49
	TRINITY_DN3534_c0_g1	GTPase-activating protein for ADP ribosylation factor	sp|Q0WQQ1|	0.5
	TRINITY_DN6529_c0_g6	ELMO domain-containing protein B	sp|Q54VR8|	0.36
Redox activity	TRINITY_DN6532_c0_g1	Protein tas	sp|P0A9T5|	0.39/0.41
	TRINITY_DN5269_c0_g3	Cytochrome P450 97B2	sp|O48921|	0.41
	TRINITY_DN4887_c0_g1	Amino oxidase domain-containing protein	tr|D8TIC9|	0.47
Glycolysis	TRINITY_DN6530_c1_g5	Endoglucanase E-4	sp|P26221|	2.07
	TRINITY_DN6530_c1_g1	Endoglucanase 1	sp|Q02934|	2.41
Surose synthesis	TRINITY_DN6326_c0_g2	4-α-glucanotransferase DPE2	sp|Q8RXD9|	2.10
Function	Protein ID	Annotation	Accession No.	FC ^b^
Energy metabolism	Maker06782	R-mandelonitrile lyase-like	sp|Q9SSM2	2.05
	Maker00854	NADH: ubiquinone oxidoreductase 30 kDa subu	sp|Q37622|	2.31
Fuction	Description	Adduct	VIP	FC ^c^
TCA	L-Malic acid	(M^−^H)^−^	2.07	3.02
	L-Asparagine	(M^+^ H)^+^	1.34	2.39
	Glyceric acid	(M^−^H)^−^	1.23	2.15
	Succinate	(M^−^H)^−^	3.66	1.67
	Cyclohexylamine	(M + CH3COO + 2H)+	1.16	1.16
TCA	Diethanolamine	(M^+^ Na)^+^	1.27	6.04
	Ribitol	(M^−^H2O^−^H)^−^	1.06	1.44
TCA	Sucrose	(M^−^H)^−^	1.54	0.84

**Table 3:** Fluctuation of molecules in *U. prolifera* on transcription and translation after 12 h treatment with high light.

Function	Transcript ID	Annotation	Accession No.	FC ^a^
Activated DNA	TRINITY_DN4269_c0_g1	DOT1 domain-containing protein	tr|A0A0D2JT68|	0.44
	TRINITY_DN5538_c0_g1	ATP-dependent DNA helicase DDX11	sp|Q6AXC6|	0.43
	TRINITY_DN4213_c0_g1	WD repeat-containing protein WRAP73	sp|Q9JM98|	0.45
	TRINITY_DN4204_c0_g1	RuvB-like 2	sp|Q9DE27|	0.44
	TRINITY_DN6524_c0_g1	Werner syndrome ATP-dependent helicase homolog	sp|O93530|	0.35
RNA transcription	TRINITY_DN6501_c2_g4	AP2-like ethylene-responsive transcription factor AIL5	sp|Q6PQQ3|	0.33/0.48
	TRINITY_DN5963_c0_g1	Transcriptional activator Myb	sp|Q08759|	0.35
	TRINITY_DN5814_c1_g1	ESF1 homolog	sp|Q76MT4|	0.39
	TRINITY_DN5608_c1_g1	DNA-directed RNA polymerase I subunit 1	sp|Q9SVY0|	0.49
	TRINITY_DN5608_c1_g1	DNA-directed RNA polymerase I subunit 1	sp|Q9SVY0|	0.49
	TRINITY_DN3823_c0_g1	RuvB-like protein 1	sp|Q9FMR9|	0.46
	TRINITY_DN1571_c0_g1	DUF2428 domain-containing protein	tr|A0A059ADU8|	0.37
RNA decay	TRINITY_DN5656_c4_g2	Tetratricopeptide repeat protein SKI3	sp|F4I3Z5|	3.46
	TRINITY_DN5703_c0_g3	Carboxypeptidase inhibitor SmCI	sp|P84875|	4.62
	TRINITY_DN4665_c0_g1	5’-nucleotidase	sp|Q61503|	0.47
	TRINITY_DN5951_c2_g4	Pseudouridine-5’-phosphate glycosidase	sp|B8F668|	0.48
	TRINITY_DN4665_c0_g1	5’-nucleotidase	sp|Q61503|	0.47
	TRINITY_DN5982_c2_g4	Cytosolic purine 5’-nucleotidase	sp|Q54XC1|	2.51
Protein synthesis	TRINITY_DN3000_c0_g1	Ribosome biogenesis protein BRX1 homolog	sp|Q8UVY2|	0.30
	TRINITY_DN5758_c1_g6	tRNA pseudouridine synthase A	sp|Q03ZL3|	0.36
	TRINITY_DN5416_c0_g1	General transcription factor 3C polypeptide 5	sp|Q9Y5Q8|	0.37
	TRINITY_DN3813_c0_g1	EEF1A lysine methyltransferase 1	sp|Q6NYP8|	0.41
	TRINITY_DN5874_c0_g2	Protein SDA1 homolog	sp|A4IIB1|	0.47
	TRINITY_DN5377_c0_g1	60S ribosomal export protein NMD3	sp|Q55BF2|	0.48
	TRINITY_DN6343_c0_g1	Eukaryotic translation initiation factor 5	sp|P55876|	0.48
	TRINITY_DN6081_c2_g7	Cytoplasmic tRNA 2-thiolation protein 2	sp|Q55EX7|	0.49
	TRINITY_DN5443_c0_g1	Protein-lysine methyltransferase METTL21D	sp|Q9H867|	0.50
	TRINITY_DN3795_c0_g1	Hybrid signal transduction histidine kinase J	sp|Q54YZ9|	0.40
	TRINITY_DN1894_c0_g1	Haloacid dehalogenase-like hydrolase domain-containing protein 3	sp|Q5E9D6|	0.43
Function	Protein ID	Annotation	Accession No.	FC ^b^
Protein synthesis	Maker02383	pre-mRNA-splicing factor ATP-dependent RNA	sp|O22899|	0.37
	Maker05539	eukaryotic translation initiation factor 5A-2	sp|P24922|	0.60
	Maker03850	DNA binding helix-turn helix protein	sp|Q9SJI8|	0.24
	Maker03627	Glutamate-cysteine ligase	sp|O23736|	0.30
	Maker04783	Polynucleotide 3’-phosphatase ZDP	sp|Q84JE8|	0.46
Function	Description	Adduct	VIP	FC ^c^
Amino acid	L-Glutamate	(M^+^ H)^+^	3.91	1.90
	L-Methionine	(M^+^ H)^+^	1.09	3.06
	L-Glutamate	(M^−^H)^−^	4.85	1.74

**Table 4:** Fluctuation of molecules in *U. prolifera* on cell signal transduction and growth after 12 h treatment with high light.

Function	Transcript ID	Annotation	Accession No.	FC ^a^
Signal transduction	TRINITY_DN5534_c0_g1	Adenylate cyclase	sp|Q01631|	0.47
	TRINITY_DN5394_c0_g2	Protein RRC1	sp|Q9C5J3|	0.44
growth	TRINITY_DN5090_c1_g6	ABC transporter G family member 31	sp|Q7PC88|	2.33
	TRINITY_DN6612_c2_g3	GPCR-type G protein 2	sp|Q0WQG8|	0.49
Ion transport	TRINITY_DN5168_c0_g1	Potassium/sodium hyperpolarization-activated cyclic nucleotide-gated channel 2	sp|O88703|	0.47
	TRINITY_DN5613_c0_g1	Sodium/calcium exchanger 3	sp|P57103|	0.35
	TRINITY_DN5780_c0_g1	Potassium voltage-gated channel subfamily H member 5	sp|Q920E3|	0.37
	TRINITY_DN6618_c1_g11	Protein detoxification	tr|E1ZHE4|	0.48
	TRINITY_DN6300_c0_g2	Solute carrier family 35 member G1	sp|Q2M3R5|	0.41
	TRINITY_DN6057_c0_g2	Sodium- and chloride-dependent GABA transporter 2	sp|P31646|	0.37
Cytoskeleton synthesis	TRINITY_DN5271_c0_g1	Kinesin-like protein KIN-4A	sp|A0A068FIK2|	0.47
	TRINITY_DN2855_c0_g1	Tubulin glycylase 3A	sp|Q9VM91|	0.38
	TRINITY_DN6187_c0_g6	Echinoderm microtubule-associated protein-like 4	sp|Q9HC35|	0.36
	TRINITY_DN5670_c0_g2	Tubulin α chain	sp|Q9ZRJ4|	0.41
	TRINITY_DN6214_c0_g1	γ-tubulin complex component 4	sp|Q9M350|	0.48
	TRINITY_DN5902_c0_g5	Tetratricopeptide repeat protein 8	sp|Q8TAM2|	0.30
	TRINITY_DN5159_c0_g1	Cilia- and flagella-associated protein 46	sp|A8ICS9|	0.32
	TRINITY_DN3562_c0_g1	Protein kintoun	sp|Q0E9G3|	0.37
	TRINITY_DN3302_c0_g1	Cilia- and flagella-associated protein 91	sp|Q95LR0|	0.40
	TRINITY_DN6507_c0_g5	Flagellar associated protein	tr|A0A0D2JXT9|	0.41
	TRINITY_DN5504_c0_g1	Intraflagellar transport protein 46	sp|A2T2 × 4|	0.43
	TRINITY_DN4300_c0_g1	Protein tilB homolog	sp|Q4R3F0|	0.46
	TRINITY_DN5700_c1_g3	Tubulin polyglutamylase TTLL4	sp|Q80UG8|	2.66
	TRINITY_DN5085_c0_g2	Dynein assembly factor 5	sp|B9EJR8|	2.00
	TRINITY_DN5844_c0_g2	Flagellar associated protein	tr|A8I7W0|	2.21
Function	Protein ID	Annotation	Accession No.	FC ^b^
Signal transduction	Maker00441	Inositol monophosphatase	sp|P54928|	0.57
	Maker05329	Calcium-dependent protein kinase 22	sp|Q9ZSA3|	0.07
	Maker04405	Mitochondrial import inner membrane translocase subunit Tim9	sp|Q9XGX8|	0.46
	Maker01722	UPF0187 protein At3g61320	sp|Q9M2D2|	1.58
	Maker04818	Vesicle-fusing ATPase	sp|Q9M0Y8|	1.61
	Maker05812	ATP-energized ABC transporter	sp|Q9C8G9|	2.63
Function	Description	Adduct	VIP	FC ^c^
Signal transduction	L-Glutamate	(M^+^ H)^+^	3.91	1.90
	Adenosine	(M^+^ CH3COO)^−^	1.58	1.86
	Succinate	(M^−^H)^−^	3.66	1.67
	Isoleucyl-Glutamate	(M^+^ H)^+^	1.16	0.71

**Table 5:** Fluctuation of molecules in *U. prolifera* on cell division, gametogenesis, and apoptosis after 12 h treatment with high light.

Function	Transcript ID	Annotation	Accession No.	FC ^a^
Cell division	TRINITY_DN5522_c0_g1	DNA mismatch repair protein MSH4	sp|F4JP48|	0.28
	TRINITY_DN6495_c2_g4	DNA replication licensing factor MCM5	sp|F4KAB8|	0.40
	TRINITY_DN6185_c0_g7	Single mybhistone 3	sp|Q6WLH4|	0.40
	TRINITY_DN6266_c0_g1	RING finger and CHY zinc finger domain-containing protein 1	sp|Q96PM5|	0.41
	TRINITY_DN2646_c0_g1	Chromosome transmission fidelity protein 18 homolog	sp|Q6NU40|	0.41
	TRINITY_DN5550_c0_g1	Origin of replication complex subunit 1A	sp|Q710E8|	0.42
	TRINITY_DN5722_c2_g2	Chromosome transmission fidelity protein 18	sp|Q9USQ1|	0.43
	TRINITY_DN5538_c0_g1	ATP-dependent DNA helicase DDX11	sp|Q6AXC6|	0.43
	TRINITY_DN5760_c1_g1	Cyclin-dependent kinase-like 4	sp|Q3TZA2|	0.44
	TRINITY_DN5922_c0_g5	DNA replication licensing factor MCM6	sp|F4KAB8|	0.45
	TRINITY_DN4213_c0_g1	WD repeat-containing protein WRAP73	sp|Q9JM98|	0.45
	TRINITY_DN6081_c2_g4	Bloom syndrome protein homolog	sp|Q9VGI8|	0.46
	TRINITY_DN6474_c4_g1	Centrosomal protein of 135 kDa	sp|Q66GS9|	0.46
	TRINITY_DN4208_c0_g1	POC1 centriolar protein homolog A	sp|Q28I85|	0.36
	TRINITY_DN6344_c2_g4	Mitotic spindle checkpoint protein MAD2	sp|Q9LU93|	0.49
	TRINITY_DN6331_c2_g8	Regulator of telomere elongation helicase 1 homolog	sp|B4PZB4|	0.39
	TRINITY_DN3611_c0_g1	Lys-63-specific deubiquitinaseBRCC36	sp|B5 × 8M4|	0.49
	TRINITY_DN6511_c0_g3	Heat shock-like 85 kDa protein	sp|P06660|	0.50
	TRINITY_DN6518_c0_g2	Histone acetyltransferase MCC1	sp|Q9M8T9|	2.87
	TRINITY_DN6346_c0_g2	Protein chromatin remodeling 24	sp|Q8W103|	2.73
	TRINITY_DN17812_c0_g1	DNA excision repair protein ERCC-6-like	sp|A2BGR3|	2.78
	TRINITY_DN6674_c1_g5	Serine/threonine-protein kinase mos	sp|Q9GRC0|	2.04
Gametogenesis	TRINITY_DN6248_c1_g1	Thioredoxin domain-containing protein 3 homolog	sp|P90666|	0.30
	TRINITY_DN5495_c0_g1	26S proteasome non-ATPase regulatory subunit 12 homolog A	sp|Q9FIB6|	0.50
	TRINITY_DN3302_c0_g1	Cilia- and flagella-associated protein 91	sp|Q95LR0|	0.40
	TRINITY_DN7873_c0_g1	C-factor	sp|P21158|	2.15
Apoptosis	TRINITY_DN4367_c0_g1	Serine/threonine-protein kinase atg1	sp|Q86CS2|	0.26
	TRINITY_DN5444_c0_g1	DnaJ homolog subfamily A member 1	sp|Q5NVI9|	0.47
	TRINITY_DN5811_c0_g1	Nucleotide-binding oligomerization domain-containing protein 1	sp|Q9Y239|	0.37
	TRINITY_DN5570_c0_g1	WD repeat-containing protein 35	sp|A6N6J5|	0.35
	TRINITY_DN6128_c2_g5	Metacaspase-1	sp|Q7XJE6|	3.14
	TRINITY_DN4163_c0_g1	Homocysteine methyltransferase	sp|Q117R7|	2.16
	TRINITY_DN5714_c3_g7	Ascorbate peroxidase	sp|Q539E5|	0.41
Function	Protein ID	Annotation	Accession No.	FC ^b^
Cell division	Maker01265	SNF1-related protein kinase regulatory subunit gamma	sp|Q8GXI9|	0.57
	Maker02620	(R)-mandelonitrile lyase	sp|O50048|	0.52
	Maker04898	DnaJ protein homolog 2	sp|P42824|	2.27

**Table 6:** Fluctuation of molecules in *U. prolifera* on resistance after 12 h treatment with high light.

Function	Transcript ID	Annotation	Accession No.	FC ^a^
Disease resistance	TRINITY_DN6596_c0_g3	disease resistance protein RGA4	sp|Q7XA39|	2.04
	TRINITY_DN6223_c1_g2	Disease resistance protein TAO1	sp|Q9FI14|	2.08/2.33
	TRINITY_DN6622_c0_g7	TMV resistance protein N	sp|Q40392|	2.30/3.03
	TRINITY_DN4211_c0_g1	DEAD-box ATP-dependent RNA helicase 50	sp|Q8GUG7|	0.22
	TRINITY_DN2527_c0_g1	17.6 kDa class I heat shock protein 3	sp|P13853|	0.27
	TRINITY_DN6083_c4_g2	Class I heat shock protein	sp|P02520|	0.48
	TRINITY_DN4570_c0_g1	Activator of 90 kDa heat shock protein ATPase homolog 1	sp|O95433|	0.50
Extradition	TRINITY_DN4877_c0_g1	Broad substrate specificity ATP-binding cassette transporter ABCG2	sp|Q7TMS5|	2.22
	TRINITY_DN6094_c2_g16	Neurotrypsin	sp|Q5G268|	2.30/2.35/2.36/2.91
Salt tolerance	TRINITY_DN6262_c1_g1	Mannitol dehydrogenase	sp|P42754|	2.66
DNA damage tolerance	TRINITY_DN6060_c1_g13	Disease resistance protein RPP5	sp|F4JNB7|	3.10
Stress adaptation	TRINITY_DN3464_c0_g1	Thiamine thiazole synthase	sp|A8J9T5|	2.42
Lipoxygenases activity	TRINITY_DN5241_c0_g1	Hydroperoxide isomerase ALOXE3	sp|Q9BYJ1|	2.36
Riboflavin synthesis	TRINITY_ DN5388 _c0_g3	Riboflavin biosynthesis protein PYRR	sp|Q9STY4|	2.99
Degradation	TRINITY_DN5268_c1_g1	Protein VMS	sp|Q04311|	2.90
	TRINITY_DN6098_c0_g2	V-type proton ATPase subunit E	sp|Q9SWE7|	2.51
Calcium depended kinase activity	TRINITY_DN5732_c1_g1	Calcium-dependent protein kinase 34	sp|Q3E9C0|	2.03
Salicylic acid synthesis	TRINITY_DN4623_c0_g2	Protein-tyrosine-phosphatase MKP1	sp|Q9C5S1|	0.41
Cell adhesion	TRINITY_DN6226_c0_g1	Lectin	sp|Q6T6H8|	2.28
DNA damage repair	TRINITY_DN4847_c0_g1	Deoxy ribodipyrimidine photo-lyase	sp|A9CJC9|	0.42
Photoprotection	TRINITY_DN4291_c0_g1	Carotene biosynthesis-related protein CBR	sp|P27516|	0.47
Oxidoreductase activity	TRINITY_DN3469_c0_g1	Molybdenum cofactor sulfurase	sp|Q4WPE6|	0.49
	TRINITY_DN6735_c7_g1	Fe2OG dioxygenase domain-containing protein	tr|D8U9C3|	0.36
	TRINITY_DN4887_c0_g1	Amino oxidase domain-containing protein	tr|D8TIC9|	0.47
Innate immune response	TRINITY_DN6599_c2_g3	NLR family CARD domain-containing protein 3	sp|Q7RTR2|	2.53/3.16
Photolyase	TRINITY_DN4847_c0_g1	Photolyase	sp|A9CJC9|	0.42
Function	Protein ID	Annotation	Accession No.	FC ^b^
Antioxidant	Maker06125	glutathione S-transferase	sp|P04907|	0.16
	Maker05392	ascorbate peroxidase	sp|P0C0L1|	0.58
	Maker05688	Peroxidase	sp|P0C0L1|	0.61
	Maker05392	Alkyl hydroperoxide reductase/thiol specificantioxidant/Mal allergen	sp|P0C0L1|	0.58
	Maker03627	Glutamate—cysteine ligase	sp|O23736|	0.30
	Maker03497	10 kDa chaperonin	sp|Q96539|	0.38
Function	Description	Adduct	VIP	FC ^c^
Resistance	γ-L-Glutamyl-L-glutamic acid	(M^+^H^−^2H2O)^+^	2.01	4.91
	L-Glutamate	(M^+^H)^+^	3.91	1.90
	D-Proline	(M^+^H)^+^	12.23	6.61
	1-Aminocyclopropanecarboxylic acid	(M^+^H)^+^	1.10	1.88
	L-Methionine	(M^+^H)^+^	1.09	3.06
	Dimethyl sulfone	(2M^+^K)^+^	1.54	1.18
	L-Asparagine	(M^+^H)^+^	1.34	2.39
	(S)-2-aminobutyric acid	(M^−^H)^−^	1.03	1.80
	2,3-Dihydroxy-3-methylbutyric acid	(M^−^H^)−^	3.42	3.73
	Ribitol	(M^−^H2O^−^H)^−^	1.06	1.44
	L-Threonate	(M^−^H)^−^	1.82	1.34
	3-Hydroxypropionic acid (β-lactic acid)	(M^+^CH3COO)^−^	1.34	1.65
	D-Lyxose	(M^−^H)^−^	1.20	1.70
	Galactinol	(M^+^CH3COO)^−^	1.38	0.82
	L-Pyroglutamic acid	(M^+^NH4)^+^	2.64	0.82
	L-Glutamine	(M^−^H)^−^	2.59	0.67

**Table 7:** Fluctuation of molecules in *U. prolifera* on cell membrane synthesis and repair after 12 h treatment with high light.

Function	Transcript ID	Annotation	Accession No.	FC ^a^
Cell membrane, cytoderm and plasmodesmata synthesis	TRINITY_DN12549_c0_g1	Enoyl-[acyl-carrier-protein] reductase [NADH]	sp|P80030|	2.23
	TRINITY_DN5887_c3_g1	sterol sensor 5-transmembrane protein	sp|Q90693	3.27
	TRINITY_DN5385_c0_g1	UDP-glucuronate 4-epimerase 1	sp|Q9M0B6|	2.19
	TRINITY_DN5951_c1_g2	UDP-glucose 6-dehydrogenase 5	sp|Q2QS13|	2.23
	TRINITY_DN5801_c0_g1	Callose synthase 12	sp|Q9ZT82|	2.62
	TRINITY_DN5550_c1_g1	Cycloartenol-C-24-methyltransferase 1	sp|Q6ZIX2|	0.45
	TRINITY_DN3918_c0_g1	Cycloeucalenol cycloisomerase	spQ9M643	0.45
tRNA synthesis	TRINITY_DN4255_c0_g1	tRNA 2’-phosphotransferase	sp|O14045|	7.56
	TRINITY_DN5185_c0_g1	Ribonuclease Z, mitochondrial	sp|Q8MKW7|	2.04
Function	Protein ID	Annotation	Accession No.	FC ^b^
Lipid biosynthesis	Maker05122	CDP-diacylglycerol-serine O-phosphatidyl transferase 2	sp|Q6I628|	1.97
	Maker05362	Adipocyte plasma membrane-associated protein	sp|Q8VWF6|	3.31
Function	Description	Adduct	VIP	FC ^c^
Cell membrane synthesis	Cis-9-palmitoleic acid	(M^−^H)^−^	1.00	2.28
	Myristic acid	(M^−^H)^−^	1.58	2.54
	Palmitic acid	(M^−^H)^−^	2.81	2.11
	α-Linolenic acid	(M^+^CH3COO)^−^	7.01	1.71
	Linoleic acid	(M^−^H)^−^	4.54	1.53
	3-Hydroxypropionic acid	(M^+^CH3COO)^−^	1.34	1.65
	4,7,10,13,16,19-Docosahexaenoic acid	M^+^	2.42	0.26
Fuction	Lipid Iond	VIP	FC^d^	
Cell membrane synthesis	DGDG (16:4/18:4) + HCOO	1.26	3.01	
	MGDG (16:0/16:4) + HCOO	1.19	0.69	
	MGMG (16:1) + HCOO	3.44	0.60	

## Data Availability

The proteome data (PXD033819) has been uploaded to the iProX database, which can be downloaded at: https://www.iprox.cn//page/SCV017.html?query=PXD033819 (accessed on 27 June 2022); The transcriptome data (SAMN28124886) has been uploaded to NCBI database, which can be downloaded at: https://www.ncbi.nlm.nih.gov/search/all/?term=SAMN28796201 (accessed on 27 June 2022).

## Supplementary Materials

The following supporting information can be downloaded at: https://www.mdpi.com/article/10.3390/biology11111563/s1, Figure S1: Gene annotation Venn diagram in transcriptome; Figure S2: Statistical table of the number of unique peptide segments in the proteome; Figure S3: Statistics of proteome data; Figure S4: Statistical of lipid subclasses and number; Table S1: Primers design of ten different genes; Table S2: QC data statistics of transcriptome; Table S3: Statistics of transcriptome assembly results; Table S4: Statistics of lipid molecules with significant differences.

## Abbreviations

DGDG	di-galactosyl diacyl glycerol
MGDG	mono-galactosyl diacyl glycerol
RNA	ribonucleic acid
DNA	deoxyribonucleic acid
qRT-PCR	quantitative real-time polymerase chain reaction
TCA	tricarboxylic acid cycle
BCA	bicinchoninic acid
PCA	principal component analysis
PLS-DA	partial least squares discriminant
OPLS-DA	orthogonal partial least-squares discriminant
PSI	photosystem I
PSII	photosystem II
FC	fold change
VIP	variable importance for the projection
TG	triglyceride
CerG1	ceramidesglycerol 1
DG	diacylglycerol
DGMG	diacylglycerol monoacylglycerol
MGMG	monogalactosyl monoacylglycerol
PG	phosphatidylglycerol
SQDG	sulfoquinovosyl diacylglycerol
ATP	adenosine triphosphate
CDPKs	calcium-dependent protein kinase
CaCA	calcium: cation antiporter

## References

[1] Zhang Y., He P., Li H., Li G., Liu J., Jiao F., Zhang J., Huo Y., Shi X., Su R. (2019). Ulva prolifera green-tide outbreaks and their environmental impact in the Yellow Sea, China. Natl. Sci. Rev..

[2] Fan S.L., Fu M.Z., Li Y. (2012). Origin and development of Huanghai (Yellow) Sea green-tides in 2009 and 2010. Bull. Mar. Sci. Miami.

[3] Ye N., Zhuang Z., Jin X., Wang Q., Zhang X., Li D., Wang H., Mao Y., Jiang Z., Li B. (2008). China is on the track tackling Enteromorpha spp forming green tide. Nat. Preced..

[4] Han W., Chen L.P., Zhang J.H., Tian X.L., Hua L., He Q., Huo Y.-Z., Yu K.-F., Shi D.-J., Ma J.-H. (2013). Seasonal variation of dominant free-floating and attached Ulva species in Rudong coastal area, China. Harmful Algae.

[5] Metaxa E., Deviller G., Pagand P., Alliaume C., Casellas C., Blancheton J.P. (2006). High rate algal pond treatment for water reuse in a marine fish recirculation system: Water purification and fish health. Aquaculture.

[6] Han H.B., Wei Z.L., Huo Y.Z., Chen Q.F., Zhang J.H., Pei-Min H.E., Ke-Feng Y.U. (2015). Effects of temperature and light intensity on the release and germination of Ulva prolifera spores/gametes. Mar. Fish..

[7] Wu H., Gao G., Zhong Z., Li X., Xu J. (2018). Physiological acclimation of the green tidal alga Ulva prolifera to a fast-changing environment. Mar. Environ. Res..

[8] Zhuo P.L., Li Y.H., Zhong J.L., Zheng M.S., Zhu W.R., Xu N.J. (2019). Comprehensive effects of exogenous salicylic acid and light on chlorophyll fluorescence parameters and photosynthetic oxygen evolution in *Ulva prolifera*. Photosynthetica. J..

[9] Cui J., Zhang J., Huo Y., Zhou L., Wu Q., Chen L., Yu K., He P. (2015). Adaptability of free-floating green tide algae in the Yellow Sea to variable temperature and light intensity. Mar. Pollut. Bull..

[10] Jia S., Wang X., Liu G., Luo D., Zhang J., Liu Y., Lin X., Liu T. (2011). Gene expression analysis of “green tide” alga *Ulva prolifera* (Chlorophyta) in China. Genes Genom..

[11] Wang Y., Liu F., Liu X., Shi S., Bi Y., Moejes F.W. (2019). Comparative transcriptome analysis of four co-occurring Ulva species for understanding the dominance of Ulva prolifera in the Yellow Sea green tides. J. Appl. Phycol..

[12] He Y., Ma Y., Du Y., Shen S. (2018). Differential gene expression for carotenoid biosynthesis in a green alga Ulva prolifera based on transcriptome analysis. BMC Genom..

[13] Fan M., Xue S., Zhi L., Wang J., Li Y., Xu N. (2018). Comparative proteomic analysis of Ulva prolifera response to high temperature stress. Proteome Sci..

[14] Bolger A.M., Lohse M., Usadel B. (2014). Trimmomatic: A flexible trimmer for Illumina sequence data. Bioinformatics.

[15] Wang L., Wang S., Li W. (2012). RSEQC: Quality control of RNA-seq experiments. Bioinformatics.

[16] Livak K.J., Schmittgen T.D. (2001). Analysis of relative gene expression data using real-time quantitative PCR and the 2 ΔΔ CT method. Methods.

[17] Fu-Liang H., Chun-Long Y., An-Que G., Yu-Lin Z., Yun-Kui L. (2014). The principle, influence factors and advantages of bicinchoninic acid method (BCA) for protein and peptide assay. Food Ferment. Ind..

[18] Wisniewski J.R. (2016). Quantitative Evaluation of Filter Aided Sample Preparation (FASP) and Multienzyme Digestion FASP Protocols. Anal. Chem..

[19] Ch A., Dc A., Xue S.A., Js B., Nx A. (2020). Primary metabolism is associated with the astaxanthin biosynthesis in the green algae Haematococcus pluvialis under light stress. Algal Res..

[20] Bilbao P., Garelli A., Diaz M., Salvador G.A., Leonardi P.I. (2020). Crosstalk between sterol and neutral lipid metabolism in the alga Haematococcus pluvialis exposed to light stress. Biochim. Biophys. Acta BBA Mol. Cell Biol. Lipids.

[21] Vavilin D.V., Ducruet J.M., Matorin D.N., Venediktov P.S., Rubin A.B. (1998). Membrane lipid peroxidation, cell viability and Photosystem II activity in the green alga Chlorella pyrenoidosa subjected to various stress conditions. Photochem. Photobiol. B Biol. J..

[22] Widzgowski J., Vogel A., Altrogge L., Pfaff J., Schoof H., Usadel B., Nedbal L., Schurr U., Pfaff C. (2020). High light induces species specific changes in the membrane lipid composition of Chlorella. Biochem. J..

[23] Eisa A., Malenica K., Schwenk Er T.S., Blt Er B. (2020). High light acclimation induces chloroplast precursor phosphorylation and reduces import efficiency. Plants J..

[24] Naradasu D., Miran W., Sharma S., Takenawa S., Soma T., Nomura N., Toyofuku M., Okamoto A. (2021). Biogenesis of outer membrane vesicles concentrates the unsaturated fatty acid of phosphatidylinositol in *Capnocytophaga ochracea*. Front. Microbiol..

[25] Yang S.P., Xie J., Cheng Y., Zhang Z., Qian Y.F. (2019). Response of Shewanella putrefaciens to low temperature regulated by membrane fluidity and fatty acid metabolism. LWT Food Sci. Technol..

[26] Mostofian B., Zhuang T., Cheng X., Nickels J. (2019). Branched-chain fatty acid content modulates structure, fluidity and phase in model microbial cell membranes. J. Phys. Chem. B.

[27] Lee A.K., Banta A.B., Wei J.H., Kiemle D.J., Feng J., Giner J.-L., Welander P.V. (2018). C-4 sterol demethylation enzymes distinguish bacterial and eukaryotic sterol synthesis. Proc. Natl. Acad. Sci. USA.

[28] Velitchkova M., Popova A., Markova T. (2001). Effect of membrane fluidity on photoinhibition of isolated thylakoids membranes at room and low temperature. Z. Für Nat. C..

[29] Yoshida K., Yokochi Y., Hisabori T. (2019). New light on chloroplast redox regulation: Molecular mechanism of protein thiol oxidation. Front. Plant Sci..

[30] Lismont C., Nordgren M., Brees C., Knoops B., Veldhoven P.V., Fransen M. (2017). Peroxisomes as modulators of cellular protein thiol oxidation: A new model system. Antioxid. Redox Signal..

[31] Basnet R., Hussain N., Shu Q. (2019). OsDGD2β is the sole digalactosyldiacylglycerol synthase gene highly expressed in anther, and its mutation confers male sterility in rice. Rice.

[32] Chng C.-P., Wang K., Ma W., Hsia K.J., Huang C. (2021). Chloroplast membrane lipid remodeling protects against dehydration by limiting membrane fusion and distortion. Plant Physiol..

[33] Hartel H., Dormann P., Benning C. (2000). DGD1-independent biosynthesis of extraplastidic galactolipids after phosphate deprivation in Arabidopsis. Proc. Natl. Acad. Sci. USA.

[34] Tong J. (2003). Progress in Study on Digalactosyl Diacylglycerol of Photosynthetic Membrane Lipids. Chin. Bull. Bot..

[35] Yang Z.L., Li L.B., Kuang T.Y. (2002). Thermal stability of oxygen evolution in photosystem II core complex in the presence of digalactosyl diacylglycerol. Chin. Sci. Bull..

[36] Hölzl G., Dörmann P. (2019). Chloroplast lipids and their biosynthesis. Annu. Rev. Plant Biol..

[37] Kang H., Jia C., Liu N., Aboagla A.A.A., Chen W., Gong W., Tang S., Hong Y. (2020). Plastid glycerol-3-phosphate acyltransferase enhanced plant growth and prokaryotic glycerolipid synthesis in Brassica napus. Int. J. Mol. Sci..

[38] Sakurai T., Kataoka K. (2007). Structure and function of type I copper in multicopper oxidases. Cell. Mol. Life Sci..

[39] Drmann P. (2010). Synthesis and Function of the Galactolipid Digalactosyl Diacylglycerol (Chapter 14). The Chloroplast.

[40] Lapointe B.E., Tenore K.R. (1981). Experimental outdoor studies with Ulva fasciata Delile. I. Interaction of light and nitrogen on nutrient uptake, growth, and biochemical composition. J. Exp. Mar. Biol. Ecol..

[41] Zhao L.S., Li K., Wang Q.M., Song X.Y., Su H.N., Xie B.B., Zhang X.-Y., Huang F., Bai-Cheng Z., Zhou B.-C. (2017). Nitrogen Starvation Impacts the Photosynthetic Performance of Porphyridium cruentum as revealed by chlorophyll a fluorescence. Sci. Rep..

[42] Brown M., Milligan A., Behrenfeld M. (2021). Photoacclimation State of Thalassiosira weissflogii is not Affected by Changes in Optical Depth Under A Fluctuating Light Regime Simulating Deep Mixing. J. Phycol..

[43] Zhao X., Zhong Y., Zhang H., Qu T., Jiang Y., Tang X., Wang Y. (2019). Cooperation between photosynthetic and antioxidant systems: An important factor in the adaptation of Ulva prolifera to abiotic factors on the sea surface. Front. Plant Sci..

[44] Zhao X., Tang X., Zhang H., Qu T., Wang Y. (2016). Photosynthetic adaptation strategy of Ulva prolifera floating on the sea surface to environmental changes. Plant Physiol. Biochem..

[45] Saco J.A., Sekida S., Mine I. (2020). Photosynthetic fluctuation accompanied by translocation of chloroplasts in Ulva conglobata (Ulvophyceae) grown under a low irradiance regime. Phycol. Res..

[46] Jiang J., Yu Y., Zheng M., Liu N., Li Y., Xu N. (2020). High light might alleviate inhibitory effects of high temperature on growth and physiological parameters of Ulva prolifera. Aquac. Res..

[47] Wang Q., Zhu B., Chen C., Yuan Z., Guo J., Yang X., Wang S., Lv Y., Liu Q., Yang B. (2021). A single nucleotide substitution of GSAM gene causes massive accumulation of glutamate 1-semialdehyde and yellow leaf phenotype in rice. Rice.

[48] Smythers A.L., Mcconnell E.W., Lewis H.C., Mubarek S.N., Hicks L.M. (2020). Photosynthetic metabolism and nitrogen reshuffling are regulated by reversible cysteine thiol oxidation following nitrogen deprivation in Chlamydomonas. Plants.

[49] Eismann A.I., Reis R.P., Silva A., Cavalcanti D.N. (2020). Ulva spp. carotenoids: Responses to environmental conditions. Algal Res..

[50] Dattolo E., Ruocco M., Brunet C., Lorenti M., Lauritano C., D’Esposito D., de Luca P., Sanges R., Mazzuca S., Procaccini G. (2014). Response of the seagrass Posidonia oceanica to different light environments: Insights from a combined molecular and photo-physiological study. Mar. Environ. Res..

[51] Hegemann P. (2009). Sensory Photoreceptors and Light Control of Flagellar Activity (Chapter 13). The Chlamydomonas Sourcebook.

[52] Neilson J., Rangsrikitphoti P., Durnford D.G. (2017). Evolution and regulation of *Bigelowiella natans* light-harvesting antenna system. J. Plant Physiol..

[53] Guan Z., Mou S., Zhang X., Xu D., Fan X., Wang Y., Wang D., Ye N. (2016). Identification and expression analysis of four light harvesting-like (Lhc) genes associated with light and desiccation stress in *Ulva linza*. J. Exp. Mar. Biol. Ecol..

[54] Todo T., Kim S.T., Hitomi K., Otoshi E., Inui T., Morioka H., Kobayashi H., Ohtsuka E., Toh H., Ikenaga M. (1997). Flavin adenine dinucleotide as a chromophore of the Xenopus (6–4)photolyase. Nucleic Acids Res..

[55] Han T.J., Kong J.A., Han Y.S., Kang S.H., Hader D.P. (2004). UV-A/blue light-induced reactivation of spore germination in UV-B irradiated Ulva pertusa (Chlorophyta). J. Phycol..

[56] Kuwano K., Abe N., Nishi Y., Seno H., Nishihara G.N., Iima M., Zachleder V. (2014). Growth and Cell Cycle of Ulva Compressa (Ulvophyceae) Under Led Illumination. J. Phycol..

[57] Jin-Jin L.I., Zhang Q., Fang Y.M. (2016). Research Progress of Xanthophyll Cycle and Its Function in Light Protection. J. Anhui Agric. Sci..

[58] Cabello-Pasini A., Aguirre-Von-Wobeser E., Figueroa F.L. (2000). Photoinhibition of photosynthesis in *Macrocystis pyrifera* (Phaeophyceae), *Chondrus crispus* (Rhodophyceae) and *Ulva lactuca* (Chlorophyceae) in outdoor culture systems. J. Photochem. Photobiol. B Biol..

[59] Eskling M., Emanuelsson A., Åkerlund H. (2001). Enzymes and Mechanisms for Violaxanthin-zeaxanthin Conversion. Regulation of Photosynthesis.

[60] Guang-Ce W., Xue-Xi T., Pei-Min H.E., Song S., Shan G., Li H., Hui W. (2016). Progress of studies on the responses of the key physiological processes including photosynthese in Ulva prolifera O.F.Müller to environmental factors. Plant Physiol. J..

[61] Carr H., Bjrk M. (2017). Parallel changes in non-photochemical quenching properties, photosynthesis and D1 levels at sudden, prolonged irradiance exposures in Ulva fasciata Delile. J. Photochem. Photobiol. B.

[62] Liu D., Ma Q., Valiela I., Anderson D.M., Keesing J.K., Gao K., Zhen Y., Sun X., Wang X. (2020). Role of C-4 carbon fixation in Ulva prolifera, the macroalga responsible for the world’s largest green tides. Commun. Biol..

[63] Shikata H., Shibata M., Ushijima T., Nakashima M., Kong S.-G., Matsuoka K., Lin C., Matsushita T. (2012). The RS domain of Arabidopsis splicing factor RRC1 is required for phytochrome B signal transduction. Plant J..

[64] Shikata H., Nakashima M., Matsuoka K., Matsushita T. (2012). Deletion of the RS domain of RRC1 impairs phytochrome B signaling in Arabidopsis. Plant Signal. Behav..

[65] Zheng Z., Gao S., Wang G. (2019). Far red light induces the expression of LHCSR to trigger nonphotochemical quenching in the intertidal green macroalgae Ulva prolifera. Algal Res..

[66] Dong L., Tu W., Liu K., Sun R., Liu C., Wang K., Yang C. (2015). The PsbS protein plays important roles in photosystem II supercomplex remodeling under elevated light conditions. J. Plant Physiol..

[67] Zhang X.W., Ye N.H., Mou S.L., Xu D., Fan X. (2013). Occurrence of the PsbS and LhcSR products in the green alga Ulva linza and their correlation with excitation pressure. Plant Physiol. Biochem..

[68] Thomas L., Marondedze C., Ederli L., Pasqualini S., Gehring C. (2013). Proteomic signatures implicate cAMP in light and temperature responses in Arabidopsis thaliana. J. Proteom..

[69] Kashith M., Keerthana B., Sriram S., Ramamurthy V., Kashith M., Keerthana B., Sriram S., Ramamurthy V. (2016). Adenylate cyclase in Arthrospira platensis responds to light through transcription. Biochem. Biophys. Res. Commun..

[70] Gordillo F., Segovia M., López-Figueroa F. (2004). Cyclic AMP levels in several macroalgae and their relation to light quantity and quality. J. Plant Physiol..

[71] Cummins J.T., Strand J.A., Vaughan B.E. (1966). Sodium transport in ulva. Biochim. Biophys. Acta BBA Biophys. Incl. Photosynth..

[72] Cummins J.T., Strand J.A., Vaughan B.E. (1969). The movement of H+ and other ions at the onser of photosynthesis in ulva. BBA Biomembr..

[73] Wong S.L., Chang J. (2000). Salinity and light effects on growth, photosynthesis, and respiration of *Grateloupia filicina* (Rhodophyta). Aquaculture.

[74] Han T., Han Y.S., Kim K.Y., Kim J.H., Shin H.W., Kain J.M., Callow J.A., Callow M.E. (2003). Influences of light and UV-B on growth and sporulation of the green alga Ulva pertusa Kjellman. J. Exp. Mar. Biol. Ecol..

[75] Kakinuma M., Coury D.A., Inagaki E., Itoh S., Yoshiura Y., Amano H. (2004). Isolation and characterization of a single-copy actin gene from a sterile mutant of *Ulva pertusa* (Ulvales, Chlorophyta). Gene.

[76] Martha M.W., Jonathon S., Jacquelyn C., Bluhm B.H., Won-Bo S., Yu J.H. (2013). Sda1, a Cys2-His2 zinc finger transcription factor, is involved in polyol metabolism and fumonisin B1 production in Fusarium verticillioides. PLoS ONE.

[77] Zimmerman Z.A., Kellogg D.R. (2001). The Sda1 protein is required for passage through start. Mol. Biol. Cell.

[78] Chastukhina I.B., Sharipova M.P., Gabdrakhmanova L.A., Balaban N.P., Safina D.R., Kostrov S.V., Rudenskaia G.N., Leshchinskaia I.B. (2004). The regulation of Bacillus intermedius glutamyl endopeptidase biosynthesis in the recombinant Bacillus subtilis strain during sporulation. Mikrobiologiia.

[79] Gomezosuna A., Calatrava V., Galvan A., Fernandez E., Llamas A. (2020). Identification of the MAPK cascade and its relationship with nitrogen metabolism in the green alga Chlamydomonas reinhardtii. Int. J. Mol. Sci..

[80] Laporte D., González A., Moenne A. (2020). Copper-Induced activation of MAPKs, CDPKs and CaMKs triggers activation of hexokinase and inhibition of pyruvate kinase leading to increased synthesis of ASC, GSH and NADPH in Ulva compressa. Front. Plant Sci..

[81] Düppre E., Schneider D. (2020). The J- and G/F-domains of the major Synechocystis DnaJ protein Sll0897 are sufficient for cell viability but not for heat resistance. FEBS Open Bio.

[82] Marka N., Kumar A., Kishor P., Rao D.M. (2020). DnaJs, the critical drivers of Hsp70s: Genome-wide screening, characterization and expression of DnaJ family genes in Sorghum bicolor. Mol. Biol. Rep..

