# Supplementary material for: Molecular Response of Ulva prolifera to Short-Term High Light Stress Revealed by a Multi-Omics Approach
Source: Biology (Basel). 2022 Oct 25;11(11):1563. doi: 10.3390/biology11111563 (PMC9687821; doi:10.3390/biology11111563)

Supporting material for

Molecular response of Ulva prolifera to short-term high light stress revealed by a multi-omics approach

Kai Gu[1+] Yuling Liu[1+], Ting Jiang[1], Chuner Cai[1,2,3*], Hui Zhao[1], Xuanhong Liu[1], Peimin He[1,2,3]

1 College of Marine Ecology and Environment, Shanghai Ocean University, Shanghai, China

2 National Demonstration Center for Experimental Fisheries Science Education (Shanghai Ocean University), Shanghai, China

3 Co-Innovation Center of Jiangsu Marine Bio-industry Technology, Lianyungang 222005, China

*Corresponding Author：Dr. Chuner Cai, College of Marine Ecology and Environment, Shanghai Ocean University, Shanghai, China-201306, E-mail: cecai@shou.edu.cn.

[+]These authors contributed equally to this work.

Content

Tables

Table S1. Primers design of ten different genes

Table S2. QC data statistics of transcriptome

Table S3. Statistics of transcriptome assembly results

Table S4. Statistics of lipid molecules with significant differences

Figure S1. Gene annotation Venn diagram in transcriptome

Figure S2. Statistical table of the number of unique peptide segments in the proteome

Figure S3. Statistics of proteome data

Figure S4. Statistical of lipid subclasses and numbers

Table S1. Primers design of ten different genes

Gene	Forward primer（5’-3’）	Reverse primer（5’-3’）	Length(bp)
18s rDNA	TGCCTAGTAAGCGCGAGTCA	AAACGATGGGCAGGGAAAC	119
TRINITY_DN5118_c0_g1	CTGACAACCTACCCATCCCC	CGTTTGTGGGCTTGCTGATG	101
TRINITY_DN5090_c1_g6	GCAGAACAACCGCACTGT	CCGACGTCTCCGAAATAGATG	120
TRINITY_DN5143_c0_g2	ACACCTTTCTTTACTTTAAACTGGT	AGCTAAATGATGGTGTGCTGT	120
TRINITY_DN3918_c0_g1	TGTGCGACAGGTGCAACAT	GGGAGCACATCAGGCTATTGA	120
TRINITY_DN6178_c1_g6	GTTCACCACGATCTTCCGCT	CCACTGTTTCAAGGGCATCG	109
TRINITY_DN6515_c0_g5	CTCCTTCAGCGCCTCATACT	CGTTCCACGCGTTTGCTC	77
TRINITY_DN4211_c0_g1	TCGGCGTTCAACAACATC	TTCCTCCTCCTGCTTCAGAG	570
TRINITY_DN4291_c0_g1	CAGAGAAGCTATCAACGCCA	CGAAGCTGGTCGAGTTCAAAAA	78
TRINITY_DN4172_c0_g2	GTATCCAGAGTTCCCGACCA	GCGCGACATACAATGACTTGG	104

Table S2. QC data statistics of transcriptome

	A1	A2	A3	C1	C2	C3
Total Reads Count(#)	41327280	50009912	41377612	42682868	39023236	40460552
Total Bases Count(bp)	5921667415	7183791842	5926713744	6133118947	5579132287	5831055701
Average Read Length(bp)	143.29	143.65	143.23	143.69	142.97	144.12
Q30 Bases Count(bp)	5710561628	6930427868	5718061370	5909513053	5382059322	5614990342
Q30 Bases Ratio(%)	96.44%	96.47%	96.48%	96.35%	96.47%	96.29%
GC Bases Count(bp)	3529771433	4279597016	3545467511	3653070737	3351054782	3511594826
GC Bases Ratio(%)	59.61%	59.57%	59.82%	59.56%	60.06%	60.22%

Note:

Total Reads Count: the number of all reads in the sample

Total Base Count: the number of bases, i.e. the amount of data

Average Read Length: mean sequence length

Q30 Base Count: the number of bases whose quality is above 30

Q30 Base Ratio: the percentage of bases whose quality is above 30

GC Base Count: the number of GC base

GC Base Ratio: The percentage of GC base

Table S3. Statistics of transcriptome assembly results

	No.	⩾500bp	⩾1000bp	N50	N90	Maximum length	Minimum length	Total length	Average length
Transcript	108031	86318	75243	5355	1804	26903	201	339698065	3144.45
Unigene	28362	13216	9470	3512	445	26903	201	39882047	1406.18

Note:

Transcript: spliced transcript sequence.

Unigene: unredundant spliced transcript sequence

N50/N90 is defined as the sequence of spliced transcripts from largest to smallest by length, with the length of the accumulated transcripts to the length of the spliced transcripts not less than 50%/90% of the total length.

Table S4. Statistics of lipid molecules with significant differences

Lipid Ion	Class	VIP	*p*-value	FC	RT-(min)
DGDG(16:4/18:4)+HCOO	DGDG	1.26	0.008	3.01	6.56
MGDG(16:0/16:4)+HCOO	MGDG	1.19	0.013	0.69	10.17
MGMG(16:1)+HCOO	MGMG	3.44	0.014	0.60	2.82
MGMG(16:2)+HCOO	MGMG	2.70	0.039	0.72	2.36
TG(18:4/16:0/18:3)+NH4	TG	1.97	0.041	0.57	18.78

Figure S1. Gene annotation Venn diagram in transcriptome

Venn diagram was used to reflect the commonality and specificity of different data sets. Different databases were represented in different colors, and the numbers represented the quantity of genes expressed specifically or in common. The overlap region represented the number of shared genes expressed in different databases, while the non-overlap region represented the number of unique genes expressed in different databases.

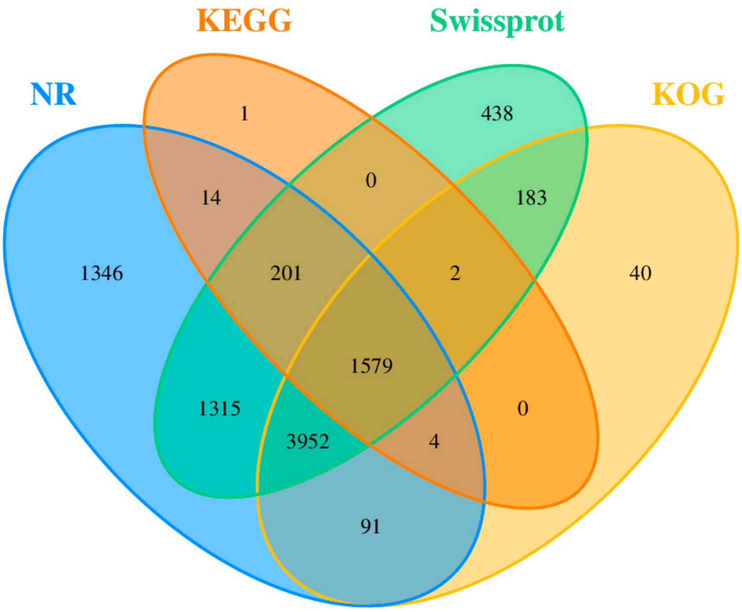

Figure S2. Statistical table of the number of unique peptide segments in the proteome

The unique peptide fragment is the protein's characteristic sequence. The horizontal axis represents the number of unique peptides, and the vertical axis represents the count of proteins used for unique peptides.

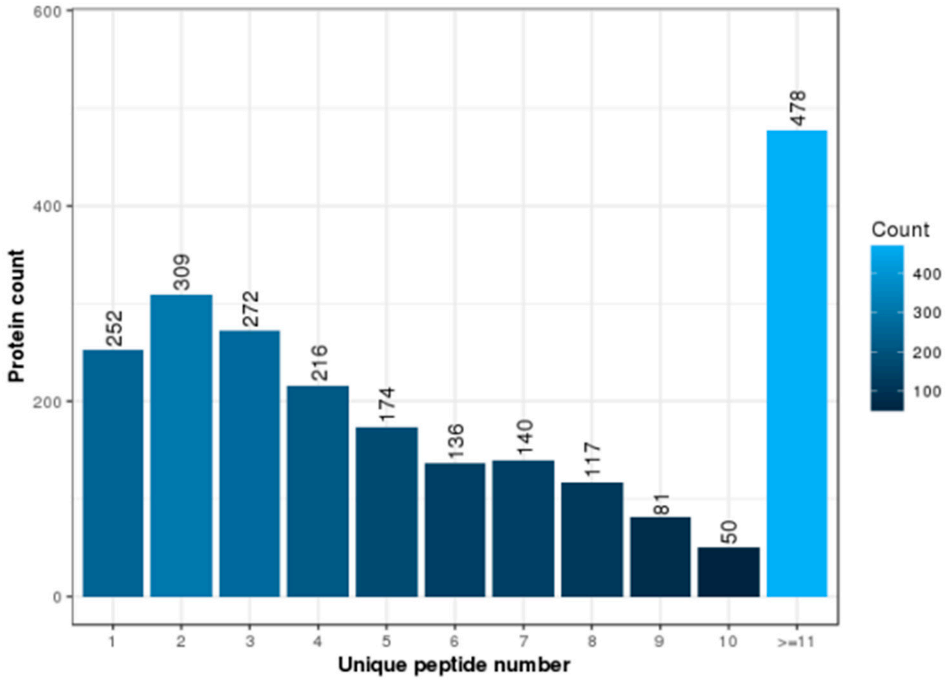

Figure S3. Statistics of proteome data

The horizontal axis represents the size of the protein, and the vertical axis represents the number of proteins of that size.

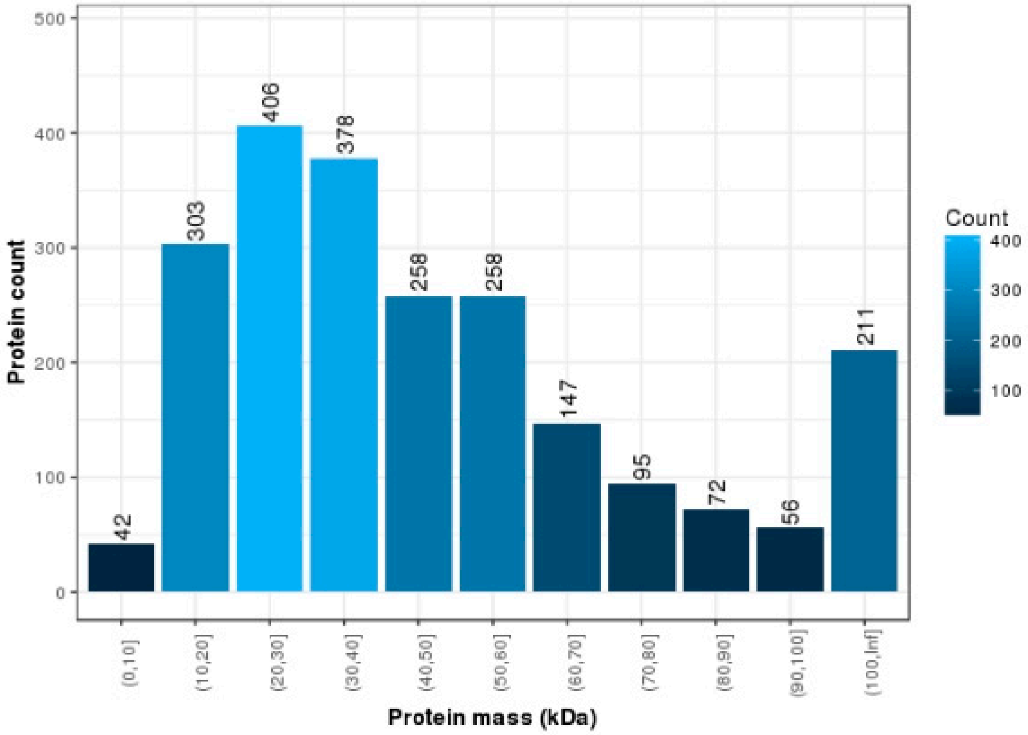

Figure S4. Statistical of lipid subclasses and number

Note: Cer: ceramides; CerG1: ceramidesglycerol 1; Co: colipase; DG: diacylglycerol; DGDG: di-galactosyl diacyl glycerol; DGMG: diacylglycerol monoacylglycerol; FA: fatty acids; LPG: lysophosphatidyl glycerol; MG: monoacylglycerol; MGDG: mono-galactosyl diacyl glycerol; MGMG: monogalactosyl monoacylglycerol; PA: phosphatidic acids ; PE: phosphatidylethanolamines; PG: phosphatidylglycerol; PI: phosphatidylinositols; PIP: phosphoinositides; PS: phosphatidylserines; So: sphingosine; SQDG: sulfoquinovosyl diacylglycerol; SQMG: sulfoquinovosyl monoglycerides; TG: triglyceride

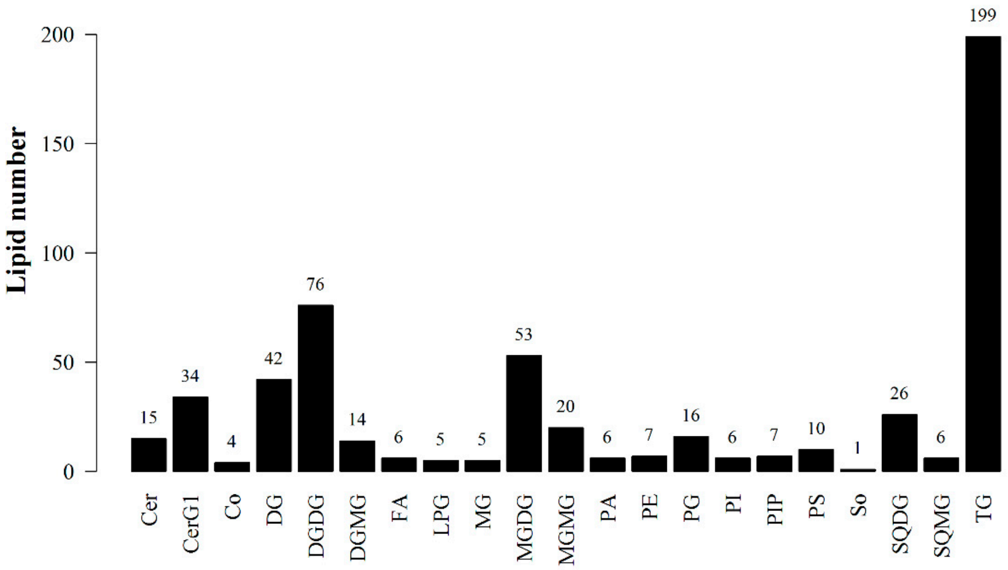

Supplement: Supplementary file 1 [file biology-11-01563-s001.zip › biology-1966882-supplementary.pdf]
